# Stille- and Ullmann-type coupling reactions catalysed by palladium supported biochar/g-C_3_N_4_-polyethyleneimine as a heterogeneous nanocatalyst

**DOI:** 10.1039/d6na00144k

**Published:** 2026-07-13

**Authors:** Maryam Nouri, Maryam Hajjami, Arash Ghorbani-Choghamarani, Zahra Siahpour

**Affiliations:** a Department of Organic Chemistry, Faculty of Chemistry and Petroleum Sciences, Bu-Ali Sina University Hamedan 6517838683 Iran m.hajjami@basu.ac.ir mhajjami@yahoo.com +988138380709 +988138282807

## Abstract

Biochar, a carbon-rich product from biomass pyrolysis, is gaining traction as a soil amendment, catalyst support, and pollutant adsorbent. This study synthesized a novel Pd@biochar/g-C_3_N_4_-PEI heterogeneous catalyst for C–C and C–N coupling reactions to produce biphenyls and aryl amines. Biochar/g-C_3_N_4_ was produced through the pyrolysis of walnut shells at 550 °C for 2 hours. Toluene diisocyanate was used to covalently bond PEI to the biochar/g-C_3_N_4_ surface, followed by the immobilization of palladium nanoparticles on this modified surface. The PEI modifier facilitates the dispersion and stabilization of the Pd nanoparticles, thereby promoting the coupling reactions. Pd@biochar/g-C_3_N_4_-PEI characterization using FT-IR, BET, SEM, TEM, TGA, ICP, EDS, and XRD confirmed the successful incorporation of PEI and Pd nanoparticles onto the biochar/g-C_3_N_4_ composite. The catalytic activity of Pd@biochar/g-C_3_N_4_-PEI was evaluated through the Stille coupling reaction and C–N coupling reactions, demonstrating high efficiency and recyclability. The catalyst exhibited excellent performance in the synthesis of various biphenyl derivatives using different aryl halides and triphenyltin chloride. Similarly, in the synthesis of aryl amines, the catalyst showed remarkable activity with various aryl halides and amines. Furthermore, the catalyst could be recycled and reused for at least five consecutive runs without significant loss of activity, highlighting its potential for sustainable applications in organic synthesis.

## Abstract 2

Biochar, a carbon-rich product from biomass pyrolysis, is gaining traction as a soil amendment, catalyst support, and pollutant adsorbent.

## Graphical abstract

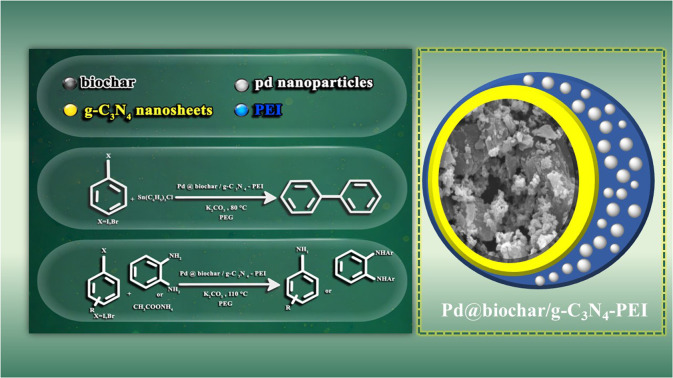

## 1 Introduction

Biochar contains significant amounts of resistant carbon and includes a variety of nutrients. The presence of micropores on the surface, combined with a large surface area and a variety of chemically active groups, provides the basis for its widespread use in carbon sequestration and soil amendment. This carbonaceous material can act as an adsorbent and neutralizer of polluting metals and can extract these harmful elements from water sources and contain them in the soil bed.^1^

Biochar is one of the important pyrolysis products that can be produced from various biomass sources and waste materials. The composition, properties, and pyrolysis conditions play a decisive role in the properties and yield of biochar.^2^ Compared to conventional activated carbon, biochar has been introduced as a new generation of economically viable and environmentally friendly carbon material that has high potential for application in various fields.^3^ To produce biochar, abundant materials are used, which are generally considered waste and are available at low cost or for free.^4^ It is possible to produce biochar from agricultural wastes such as wood, peanut shells, hazelnuts, and wheat straw through a slow pyrolysis process carried out at temperatures of 300 to 900 degrees Celsius in an oxygen-free atmosphere.^5^ The properties of biochar are particularly influenced by various factors such as the composition of the raw materials, pyrolysis conditions, and biochar activation parameters.

Biochar is a stable carbonaceous material obtained by controlled heating of plant sources, providing the possibility of replacing conventional fuels in power generation plants. Biochar has a porous structure with a high specific surface area and chemical functional groups. These properties have made it useful in the fields of catalysis of various processes, catalyst support, and effective and inexpensive adsorption of gaseous pollutants such as SO_2_, NO_*x*_, mercury, CO_2_, and volatile organic compounds from industrial flue gases.^6^ Among the surface properties of biochar that directly or indirectly affect its functional performance,^7,8^ oxygen containing functional groups (OCFGs), mainly composed of carbon (C) as the most abundant element and oxygen (O) as the second major element,^9^ are considered critical performance indicators and have great potential for pollutant removal, catalysis, and electrochemical applications.^10,11^

When pyrolysis of biological materials is carried out under continuous heating in an oxygen-free or oxygen-deficient environment, the study of the degradation processes of the three key components (including cellulose, hemicellulose, and lignin) is often considered a fundamental path to understanding the complex mechanism of biomass thermal decomposition. Common oxygenated functional groups (OCFG) in biochar include hydroxyl (–OH), carboxyl (–COOH), ether (C–O–C), carbonyl (C=O), alkoxyl (C–O), and aldehyde (–CHO), of which –OH and –COOH groups are the most abundant in the biochar structure.^12–14^

Given the remarkable properties of biochar as a catalyst support, researchers have sought to enhance its performance through surface modification with advanced materials. One of the important two-dimensional nanomaterials is graphitic carbon nitride (g-C_3_N_4_), which is known as a valuable material for membrane applications due to its controllable structure and suitable stability properties.^15^ Carbon-based compounds, particularly g-C_3_N_4_, offer several advantages compared to conventional semiconductor sonocatalysts, exhibiting significant physicochemical resistance due to their graphene-like layered structure.^16^ In addition, simple and cost-effective synthesis, a suitable energy range, and light absorption capacity in the visible region are other advantages of this material.^17^ Carbon-based materials, including g-C_3_N_4_ and biochar, which are derived from natural and existing elements, have garnered increasing interest from researchers due to their high potential in environmental remediation and improvement techniques.^18^ Therefore, biochar modified with g-C_3_N_4_ has found widespread application as a carrier substrate for precious metals in various fields. For instance, Hajjami *et al.* (2025) reported the successful synthesis of a Pd–arginine complex supported on walnut shell-derived biochar as an efficient and recyclable nanocatalyst for both C–C and C–N cross-coupling reactions, demonstrating excellent activity and reusability under mild conditions.^19^ In a recent study by Liu *et al.* (2024), a silver-modified biochar and graphitic carbon nitride (Ag-CN@BC) composite was developed to enhance the photocatalytic degradation of BPA under persulfate (PDS) activation. This modification improved visible light absorption and charge separation, while the biochar acted as a carrier to enhance catalyst stability, highlighting the importance of material design in improving photocatalytic performance.^20^ In another study, Alekasir *et al.* (2024) synthesized biochar from biomass waste and modified its surface to immobilize a novel copper complex, producing a reusable nanocatalyst for the homoselective synthesis of tetrazoles. Their study demonstrates the effectiveness of modified biochar as a stable catalyst support in organic reactions.^21^

In addition to environmental applications, biochar-based catalysts have unique properties that open up new avenues in organic synthesis, particularly in the formation of carbon-heteroatom bonds. Heterocyclic compounds, especially nitrogen-containing ones, are of great importance because they are abundantly present in diverse natural materials as well as in synthetic organic molecules with important biological activities.^22^ The formation of carbon-nitrogen (C–N) bonds is crucial because it allows nitrogen to be incorporated into the structure of organic molecules^23^ and represents a significant challenge for medicinal chemists.^24^ The purposeful addition of an amino functional group was first proposed by Ullmann and Goldberg more than 100 years ago.^25,26^ Current methods for C–N bond generation are based on activated starting materials such as (pseudo)aryl and (hetero)aryl halides for reaction with amine compounds.^22^ However, the production of C–N bonds in a mild and low-cost reaction environment remains a significant challenge.^23^

Similarly, the production of carbon–carbon (C–C) bonds has remained a vital tool in the toolkit of organic synthesis chemists since its inception in the 19th century.^27^ A fundamental breakthrough in C–C cross-coupling was achieved through the use of highly reactive ligand-containing palladium catalysts to react with aryl halides, aryl pseudohalides, or vinyl halides in the presence of organometallic compounds (B, Mg, Si, Zn, and Sn) or alkenes.^28^ Aromatic substitutions are a very important group of reactions in the fine chemical and pharmaceutical industries. The use of palladium as a catalyst in such processes is common during the research and product development stages, and extensive efforts have been devoted to the design of supported and nanostructured Pd catalysts to enhance activity, stability, and recyclability^29–31^ Its attractive characteristics include strong selectivity, compatibility with a wide range of functional groups, application without protecting groups and strong bases, and application in the final steps of total synthesis.^32^

In the field of organic chemistry, the arylation Stille coupling reaction is considered one of the most prominent and reliable palladium-catalyzed cross-coupling techniques for the formation of C–C bonds. This process uses tin-containing compounds and is carried out under mild operating conditions by employing small amounts of palladium complexes. The resistance of organotin agents to water and oxygen gives this method a significant advantage over other palladium-based coupling techniques. This method is effective in the preparation of valuable organic compounds and is usually superior in the production of complex structures, including biological compounds.^33^

Despite significant advances in palladium-catalyzed cross-coupling reactions, the high cost of palladium catalysts and their difficult recovery from reaction mixtures remain major challenges for large-scale applications. The integration of palladium with biochar/g-C_3_N_4_ supports offers a promising solution by combining the high surface area and functional groups of biochar with g-C_3_N_4_. Therefore, this study aims to synthesize a palladium-supported biochar/g-C_3_N_4_ nanocomposite and evaluate its catalytic performance in both C–C (Stille-type) and C–N (Ullmann-type) cross-coupling reactions, with particular emphasis on recyclability and stability under mild reaction conditions.

## 2 Experimental

X-ray diffraction (XRD) patterns were recorded on a Philips PW1730 diffractometer (Philips, The Netherlands). Thermogravimetric analysis (TGA) was performed using a TA Instruments Q600 analyzer (TA Instruments, USA). Fourier-transform infrared (FT-IR) spectra were obtained on a PerkinElmer spectrometer (Spectrum version 10.03.06). Transmission electron microscopy (TEM) images were acquired using a Philips EM 208S microscope. The specific surface area and pore structure were determined by Brunauer–Emmett–Teller (BET) analysis using a Mini II analyzer. Field emission scanning electron microscopy (FESEM) coupled with energy-dispersive X-ray spectroscopy (EDS) and elemental mapping (MAPP) was conducted on an FEI Quanta instrument. Inductively coupled plasma (ICP) analysis was performed using a Varian 730-ES instrument. Nuclear magnetic resonance (NMR) spectra were recorded on a Bruker AVANCE III (300 MHz) and Bruker DRX500 (500 MHz, Bruker, Germany). Gas chromatograph-mass spectrometer (GC-MS) was performed using Scion Netherlands – model 456-SQ, with the CPSil 5 Capillary Column (25 m × 0.25 mm × 0.25 macrom).

### 2.1 Preparation of biochar

Initially, walnut shells were selected and washed carefully with deionized water to remove any impurities. They were then dried in sunlight for three days. After drying, the shells were gently crushed and passed through a sieve with a 1 mm mesh diameter to obtain uniform particles. Subsequently, biochar was prepared from the air-dried walnut shells through a pyrolysis process under an oxygen-free atmosphere for two hours at 600 °C with a heating rate of 5 °C min^−1^ in a furnace.

### 2.2 Biochar/g-C_3_N_4_ composite synthesis

Initially, 5 g of urea was stirred in 5 mL of distilled water using a magnetic hot stirrer for 30 minutes. Then, 5 g of biochar obtained from walnut shells was added to this solution, and the mixture was stirred for another 30 minutes. Subsequently, this mixture was heated in a furnace at 550 °C with a heating rate of 5 °C min^−1^ for two hours. Finally, after drying at room temperature, the g-C_3_N_4_/biochar composite was obtained.

### 2.3 g-C_3_N_4_/biochar-PEI synthesis

The biochar/g-C_3_N_4_ composite was uniformly dispersed in 30 mL of toluene using an ultrasonic bath. Then, 2.5 mL of toluene diisocyanate (TDI) was added to the mixture, and the reaction was carried out at 25 °C for 30 minutes until the drying stage occurred.

One gram of the obtained solid was stirred in 25 mL of toluene at 25 °C for 40 minutes. Subsequently, the reaction mixture was filtered, and the solid was washed with toluene and dried at 70 °C for 30 minutes.

1 g of the dried solid was dispersed in 25 mL of isopropanol. Finally, 1 gram of polyethyleneimine (PEI, 50 wt%) was added to the mixture along with 10 mL of water and stirred continuously for 2 h at 50 °C. Finally, the biochar/g-C_3_N_4_-PEI product was purified using several washing steps with distilled water and dried for 24 hours at room temperature.

### 2.4 Pd@biochar/g-C_3_N_4_-PEI synthesis

The biochar/g-C_3_N_4_-PEI product (1 g) was dispersed in 25 mL of ethanol for 15 minutes, then 0.25 g of palladium acetate was added, and the mixture was stirred under reflux conditions at 80 °C for 20 hours. Finally, 0.3 g of sodium borohydride was added to the mixture and the reaction was continued for another 4 hours. Finally, the obtained product was filtered, washed with water and ethanol, respectively, and dried at ambient temperature.

### 2.5 General procedure for C–C coupling reactions

A mixture of 1 mmol aryl halides, 0.5 mmol triphenyltin chloride, and 3 mmol potassium carbonate in 2 mL polyethylene glycol-400 (PEG-400) was combined with 0.004 g of Pd@biochar/g-C_3_N_4_-PEI (containing 0.86 mol% Pd) catalyst in a sealed reaction vessel. The mixture was stirred at 80 °C until the reaction was complete. After cooling to room temperature, the mixture was diluted with 30 mL water, extracted with ethyl acetate (3 × 10 mL), and dried over sodium sulfate. Evaporation of the solvent yielded pure biphenyl derivatives in high yields. The use of PEG-400 as a solvent makes this process more environmentally friendly.

### 2.6 General procedure for the C–N coupling reaction

A mixture of aryl halides (1 mmol), 1,2-benzenediamine (0.5 mmol)/ammonium acetate (1 mmol), and potassium carbonate (3 mmol) in polyethylene glycol-400 (PEG-400, 3 mL) was reacted with 0.006 g of Pd@biochar/g-C_3_N_4_-PEI catalyst (containing 1.29 mol% Pd). The sealed reaction vessel was stirred at 110 °C until completion. After cooling to room temperature, the mixture was diluted with 30 mL of water and extracted with ethyl acetate (3 × 10 mL). The combined organic phases were dried over anhydrous Na_2_SO_4_, and the solvent was evaporated, yielding pure products in good to excellent yields. The crude product was purified by planar chromatography on silica gel, eluting with a gradient of ethyl acetate in hexanes.

### 2.7 Selected NMR data

#### 2.7.1. 1,1′Byphenyl

^1^H NMR (500 MHz, DMSO) *δ* 7.68–7.60 (m, 2H), 7.46 (t, *J* = 7.7 Hz, 2H), 7.39–7.32 (m, 1H). ^13^C NMR (126 MHz, DMSO) *δ* 140.19, 128.92, 127.41, 126.68.

#### 2.7.2. 4-Methoxy-1,1′byphenyl

^1^H NMR (500 MHz, DMSO) *δ* 7.64–7.54 (m, 4H), 7.45–7.38 (m, 2H), 7.32–7.25 (m, 1H), 7.04–6.96 (m, 2H), 3.78 (s, 3H). ^13^C NMR (126 MHz, DMSO) *δ* 158.9, 139.8, 132.5, 128.8, 127.7, 126.7, 126.1, 114.3, 55.1.

#### 2.7.3. 4-Methyl-1,1′byphenyl

^1^H NMR (250 MHz, DMSO) *δ* 7.61 (d, *J* = 10.4 Hz, 2H), 7.44 (dd, *J* = 9.1, 2.0 Hz, 1H), 7.24 (d, *J* = 8.2 Hz, 2H), 2.31 (s, 3H). ^13^C NMR (63 MHz, DMSO) *δ* 140.5, 137.7, 137.1, 129.9, 129.3, 127.5, 127.3, 126.9, 126.8, 21.0.

#### 2.7.4. Aniline

^1^H NMR (300 MHz, DMSO) *δ* 7.72–7.63 (m, 3H), 7.53–7.44 (m, 3H), 7.42–7.34 (m, 1H). ^13^C NMR (75 MHz, DMSO) *δ* 140.6, 129.4, 127.9, 127.1.

#### 2.7.5. *N*,*N*-bis(1,1′byphenyl)-1,2-benzendiamine

^1^H NMR (300 MHz, DMSO) *δ* 7.67 (dd, *J* = 8.1, 1.6 Hz, 3H), 7.13 (dd, *J* = 8.4, 6.9, 1.6 Hz, 3H), 6.84 (dd, *J* = 8.3, 1.4 Hz, 3H), 6.60 (dd, *J* = 8.3, 7.0, 1.4 Hz, 3H), 6.41 (s, 4H). ^13^C NMR (75 MHz, DMSO) *δ* 145.8, 140.6, 137.1, 131.7, 122.2, 117.1, 116.0.

## 3 Results and discussion

### 3.1 Catalyst characterization

After synthesizing the catalyst, it was characterized and evaluated using various techniques, such as FTIR, SEM, EDX, TGA, ICP, BET, TEM, and XRD.

#### 3.1.1. SEM analysis

**Fig. 1:**
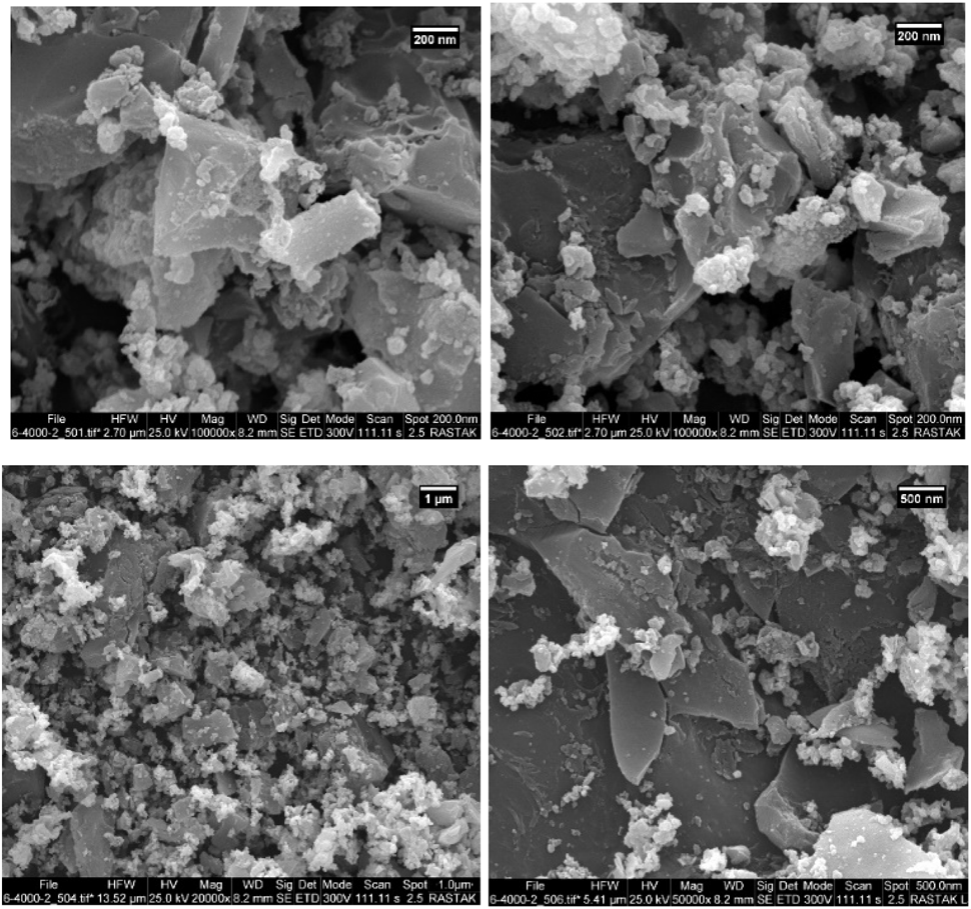
SEM image of the Pd@biochar/g-C_3_N_4_-PEI catalyst.

Based on the scanning electron microscopy (SEM) analysis of the final Pd@biochar/g-C_3_N_4_-PEI catalyst images presented in Fig. 1. The shiny particles dispersed on the surface are interpreted as palladium nanoparticles and indicate effective loading. The images demonstrate features at the nanometer scale.

#### 3.1.2. TEM analysis

**Fig. 2:**
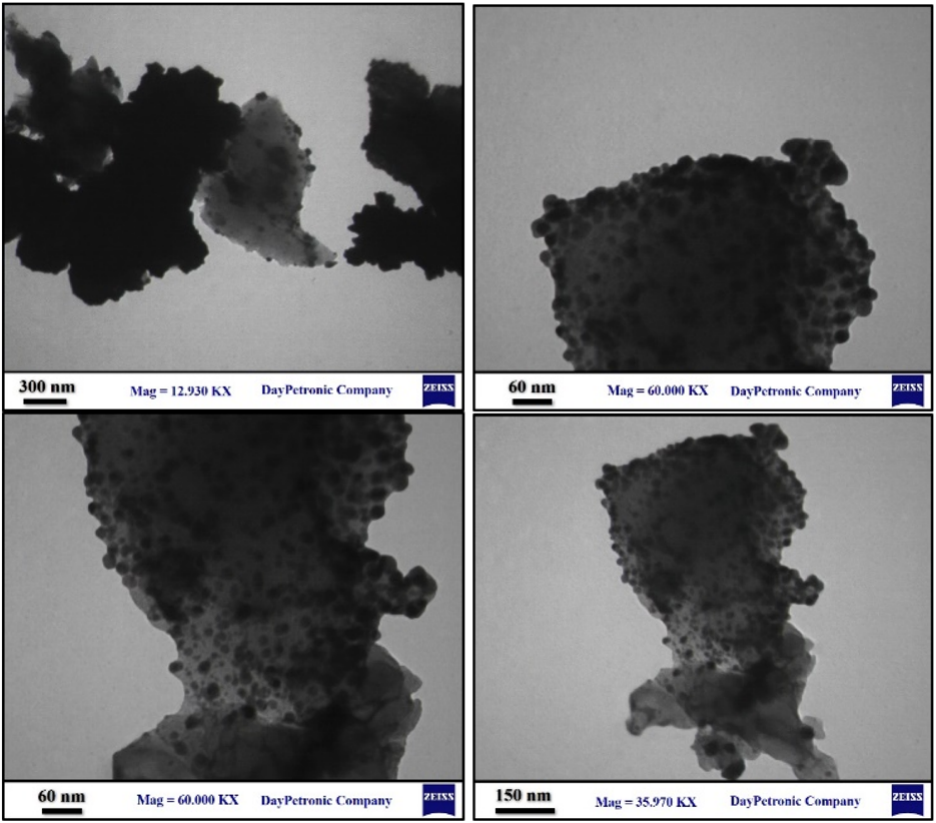
TEM image of the Pd@biochar/g-C_3_N_4_-PEI catalyst.

Transmission electron microscopy (TEM) is a powerful technique for nanoscale material imaging. TEM works by transmitting an electron beam through an ultrathin specimen; the resulting electron–atom interactions generate a high-resolution, high-magnification image, revealing details down to the atomic level. Transmission electron microscopy (TEM) reveals that the Pd@biochar/g-C_3_N_4_-PEI catalyst particles are typically less than 100 nm in size (Fig. 2). TEM images show well-dispersed Pd nanoparticles (dark spots) on the biochar/g-C_3_N_4_-PEI support, enabling detailed microstructure and composition analysis. The g-C_3_N_4_ nanosheets offer a large surface area for Pd deposition, while PEI enhances the interaction between biochar and g-C_3_N_4_. The biochar support stabilizes the nanoparticles and prevents agglomeration, crucial for catalytic activity.

#### 3.1.3. XRD analysis

**Fig. 3:**
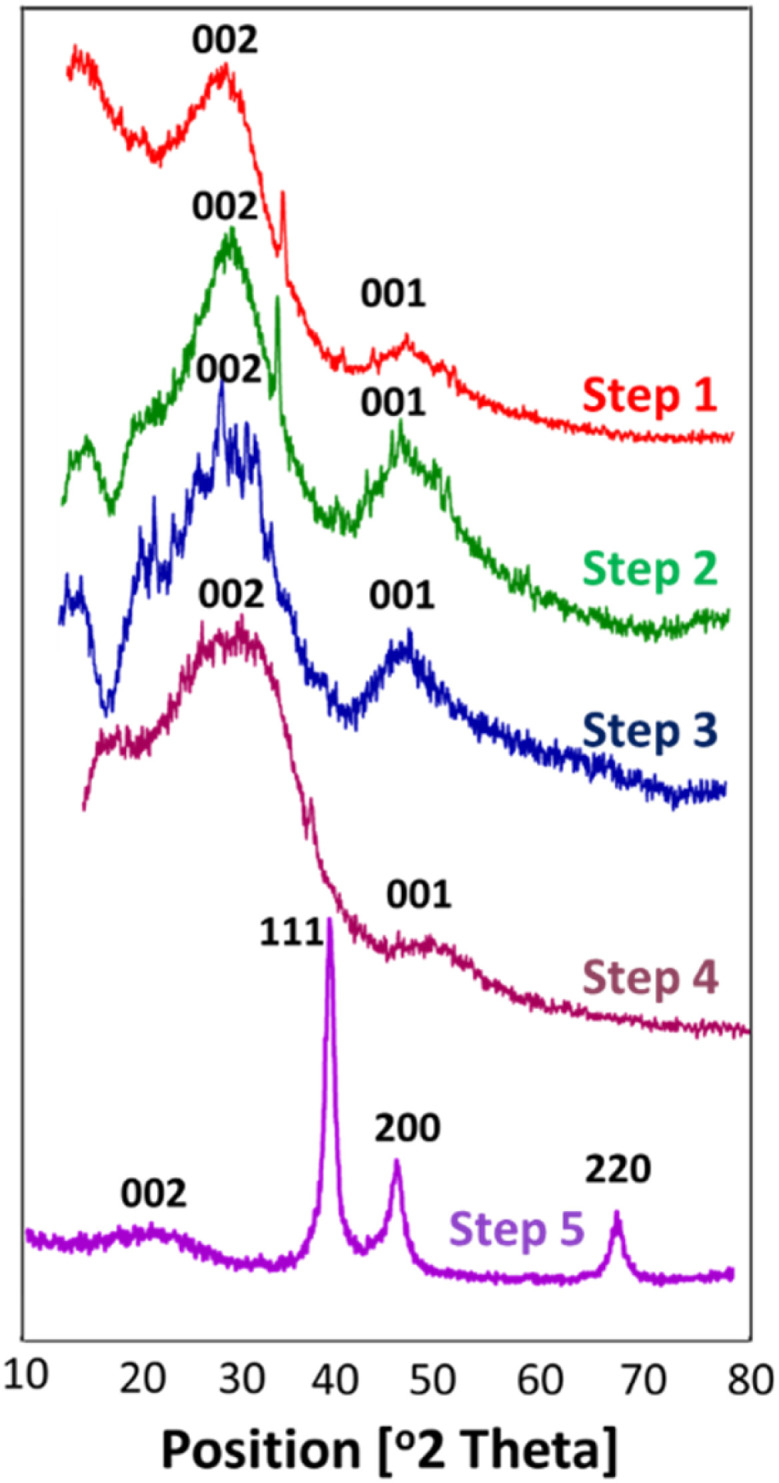
XRD patterns of biochar (step 1), biochar/g-C_3_N_4_ (step 2), biochar/g-C_3_N_4_/TDI (step 3), biochar/g-C_3_N_4_-PEI (step 4), and Pd@biochar/g-C_3_N_4_-PEI (step 5).

The XRD patterns of the synthesized steps at different stages of catalyst preparation are shown in Fig. 3. The XRD pattern of the walnut shell biochar showed two broad peaks at approximately 2*θ* = 23° and 43°, which are related to the (002) and (100) planes of amorphous carbon, respectively, confirming the disordered carbon structure, which is characteristic of biochar prepared from biomass produced at a pyrolysis temperature of 600 °C.^34^ After the formation of the biochar/g-C_3_N_4_ composite, two characteristic peaks appeared at approximately 2*θ* = 13° and 27°, which are attributed to the (100) and (002) planes, respectively, confirming the successful formation of g-C_3_N_4_ on the biochar surface.^35^ After surface modification with TDI, the characteristic peaks of g-C_3_N_4_ were maintained, while a slight decrease in their intensity was observed, which was attributed to the amorphous coating of TDI molecules on the composite surface. After functionalization with PEI, the g-C_3_N_4_ peaks became significantly weaker and a broad and dominant peak appeared at approximately 2*θ* = 20–23°, confirming the successful attachment of PEI to the composite surface *via* the TDI linker.^36^ Finally, the XRD pattern of the Pd@biochar/g-C_3_N_4_-PEI catalyst showed distinct peaks at 2*θ* = 40°, 46°, and 68°, which correspond to the (111), (200), and (220) crystal planes of metallic palladium,^37^ respectively, confirming the successful formation and uniform distribution of palladium nanoparticles on the composite surface.

#### 3.1.4. BET analysis (surface area and porosity analysis)

**Fig. 4:**
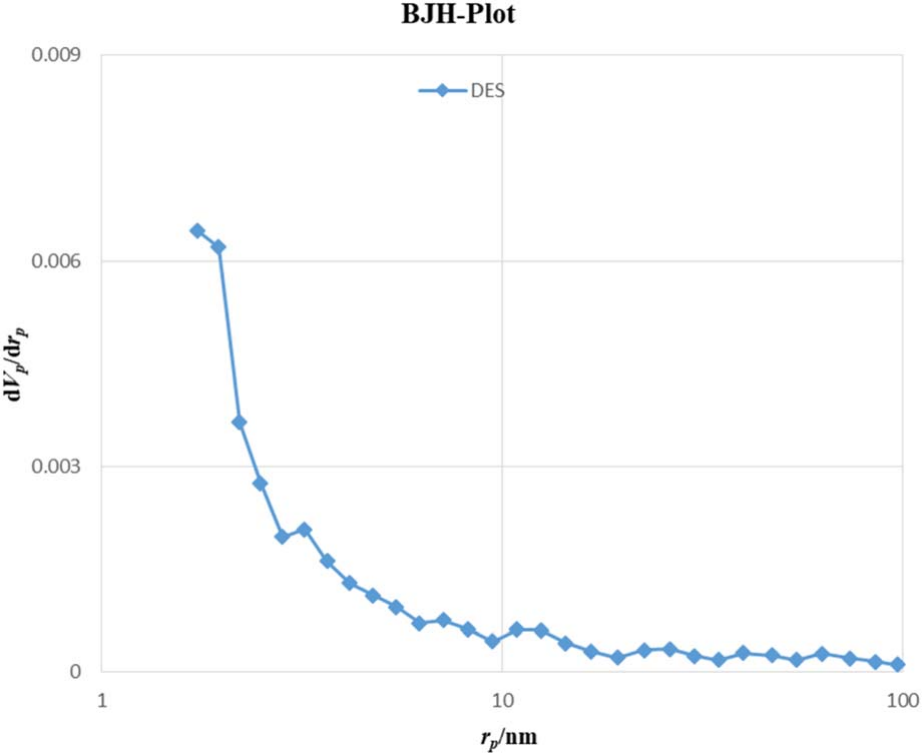
BJH adsorption d*V*/d*r* pore volume distribution for Pd@biochar/g-C_3_N_4_-PEI nanoparticles.

**Fig. 5:**
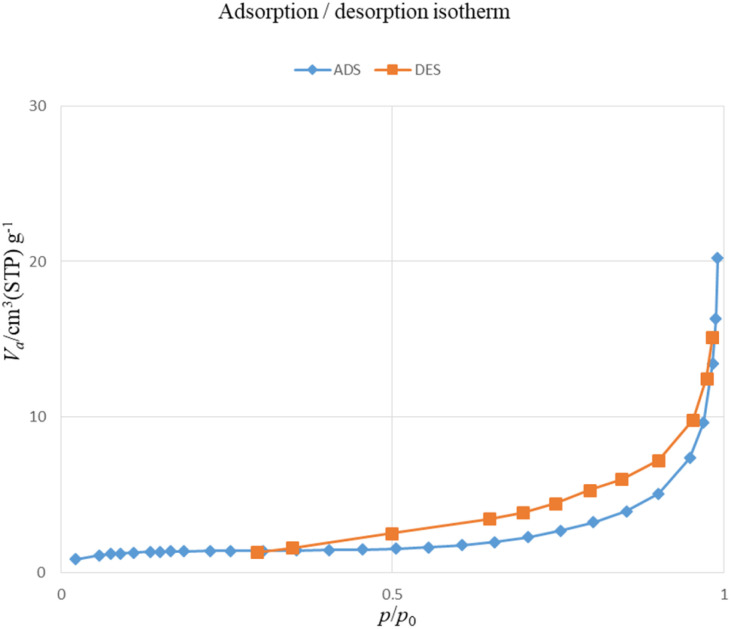
Nitrogen adsorption–desorption isotherms for Pd@biochar/g-C_3_N_4_-PEI nanoparticles.

The Brunauer–Emmett–Teller (BET) method is a fundamental technique for measuring the specific surface area of porous materials, including solids and powders. This analysis is crucial for adsorption process research, particularly when dealing with materials possessing porous structures. While accurate under ideal conditions, the BET method's precision can be compromised by nanoparticle aggregation (masking internal surfaces), violations of underlying assumptions, calibration errors, and flawed sample preparation, all of which can lead to underestimations of surface area.^38^ Adsorption characterization of Pd@biochar/g-C_3_N_4_-PEI was analyzed through N_2_ adsorption at 77.35 K over a relative pressure (*P*/*P*_0_) range of 0.0–1. The Pd@biochar/g-C_3_N_4_-PEI sample pyrolyzed at 600 °C for 2 hours showed a specific surface area of 4.95 m^2^ g^−1^ acting as a heterogeneous catalyst for C–C and C–N coupling reactions. The BJH method was employed to determine the pore size distribution of the adsorbent. Fig. 4 illustrates the pore size distribution curve for the Pd@biochar/g-C_3_N_4_-PEI nanoparticle sample. The curve depicts pore size distribution in the micropore (<2 nm), mesopore (2–50 nm), and macropore (>50 nm) regimes. The analyzed sample displays sizes ranging from 1.7 to 100 nm, distributed mainly in the mesoporous regime. The nitrogen adsorption–desorption isotherm of Pd@biochar/g-C_3_N_4_-PEI nanoparticles is depicted in Fig. 5, showing that the adsorbent exhibits a type IV isotherm with an H3 hysteresis loop across a broad *P*/*P*_0_ range of 0.02 to 0.99, indicative of a mesoporous structure.^39^

#### 3.1.5. FT-IR spectra

**Fig. 6:**
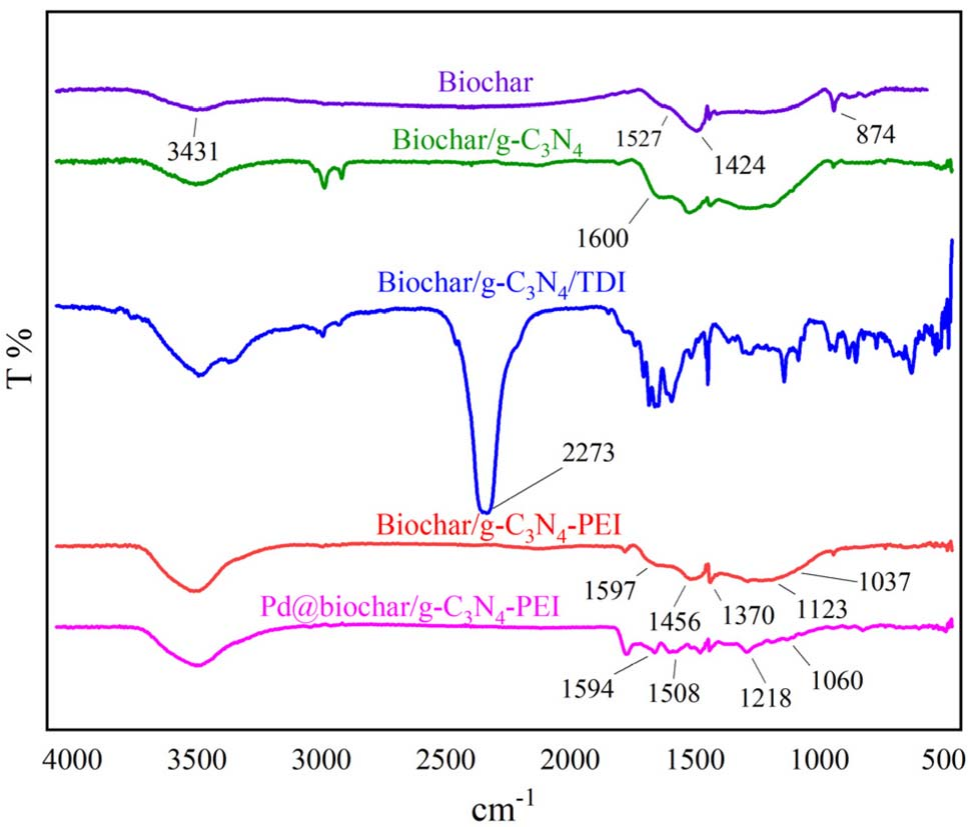
FT-IR spectrum of biochar (purple), biochar/g-C_3_N_4_ (green), biochar/g-C_3_N_4_/TDI (blue), biochar/g-C_3_N_4_-PEI (red), and Pd@biochar/g-C_3_N_4_-PEI (pink).

Fig. 6 presents the FT-IR spectrum (400–4000 cm^−1^) of the Pd@biochar/g-C_3_N_4_-PEI nanocatalyst, illustrating the functional groups on the surface. Biochar FT-IR data show a broad absorption peak at 3431 cm^−1^, indicating –OH stretching vibrations, and peaks at 1527 cm^−1^ and 1424 cm^−1^, attributed to C=C stretching. The weak peak at 874 cm^−1^ indicates C–H stretching vibrations. The FT-IR spectrum of biochar/g-C_3_N_4_ shows a C=N stretch at 1600 cm^−1^. In the biochar/g-C_3_N_4_@TDI spectrum, diisocyanate groups exhibit characteristic absorptions at 2273 cm^−1^. In biochar/g-C_3_N_4_@PEI, peaks at 1370 cm^−1^ and 1037–1123 cm^−1^ indicate C–N stretching vibrations, while the 1456 cm^−1^ band corresponds to C–H bonds. Bands at 1597 and 1456 cm^−1^, attributed to N–H vibrations of primary and secondary amino groups, shift to lower wavenumbers in Pd@biochar/g-C_3_N_4_-PEI, confirming Pd complex formation and a strong interaction between Pd and PEI amino groups, which is crucial for enhanced catalytic activity. Biochar provides stable support for g-C_3_N_4_ and PEI, preventing aggregation and improving Pd nanoparticle dispersion. This synergistic effect of biochar, g-C_3_N_4_, PEI, and Pd nanoparticles results in a highly efficient and stable catalyst.

#### 3.1.6. TGA analysis

##### 3.1.6.1 TGA analysis of biochar

**Fig. 7:**
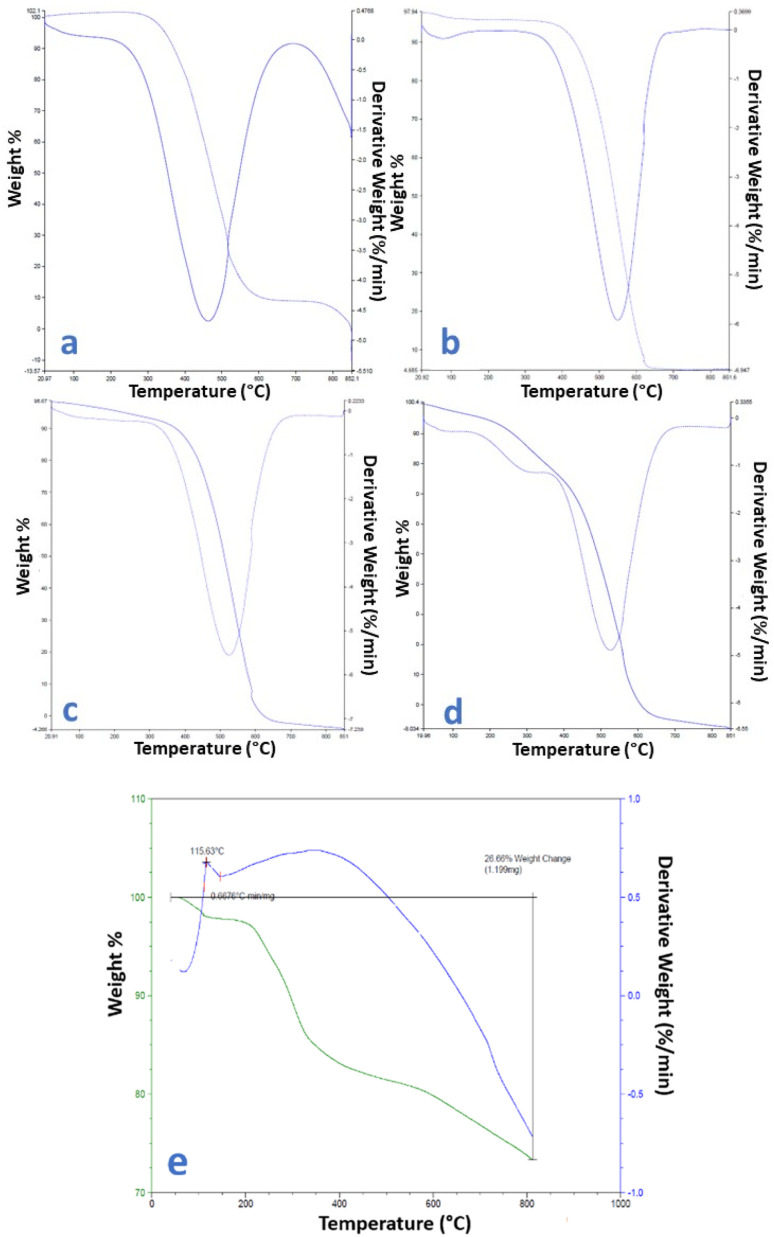
The TGA diagram of biochar (a), biochar/g-C_3_N_4_ (b), biochar/g-C_3_N_4_/TDI (c), biochar/g-C_3_N_4_-PEI (d), and Pd@biochar/g-C_3_N_4_-PEI (e).

Qualitative determination of the organic groups grafted on the biochar surface was studied by TGA analysis with a heating rate of 10 °C min^−1^ in the range 30–1000 °C. The TGA curve of biochar is shown in Fig. 4. The TGA diagram reveals two stages of weight loss. The gradual 70% weight loss between 300 and 500 °C corresponds to the elimination of hydroxyl and acidic functional groups on the biochar surface (Fig. 7, diagram a). The broad peak in derivative thermogravimetric (TGA) analysis between 400 and 500 °C indicates mass loss from the degradation and decomposition of organic matter and carbon, likely converting to CO_2_, CO, CH_4_, and other minor substances.^40^

##### 3.1.6.2 TGA analysis of biochar/g-C_3_N_4_

Qualitative determination of the organic groups grafted on the biochar surface was studied by TGA analysis with a heating rate of 10 °C min^−1^ in the range 30–1000 °C. The TGA curve of biochar/g-C_3_N_4_ is shown in Fig. 7, diagram b. The TGA diagram reveals two stages of weight loss. Initially, a 3–4% weight loss below 100 °C signifies moisture removal. Subsequently, a gradual 50% weight loss between 400 and 550 °C corresponds to the elimination of hydroxyl and acidic functional groups on the biochar surface and the degradation of triazine rings, confirming the formation of g-C_3_N_4_ with biochar^41^ (Fig. 7, diagram b).

##### 3.1.6.3 TGA analysis of biochar/g-C_3_N_4_/TDI

Qualitative determination of the organic groups grafted on the biochar surface was studied by TGA analysis with a heating rate of 10 °C min^−1^ in the range 30–1000 °C. The TGA curve of biochar/g-C_3_N_4_/TDI is shown in Fig. 7, diagram c. The TGA diagram shows two stages of weight loss. The weight loss of about 3% below 200 °C is related to the evaporation of solvents and hydroxyl groups on the surface of biochar.^39^ The organic contents decomposed at 300–500 °C, resulting in a decrease in the mass of about 40% at this temperature. Finally, the compounds decomposed in the temperature range of 500–600 °C.

##### 3.1.6.4 TGA analysis of biochar/g-C_3_N_4_/PEI

Qualitative determination of the organic groups grafted on the biochar surface was performed by TGA analysis with a heating rate of 10 °C min^−1^ in the range of 30–1000 °C. The TGA curve of biochar/g-C_3_N_4_/PEI c is shown in Fig. 7, diagram d. The TGA diagram shows three stages of weight loss. The weight loss of about 6% below 200 °C is related to the evaporation of solvents and hydroxyl groups on the surface of biochar.^39^ The organic contents decomposed at 200–400 °C, resulting in a decrease in the mass of about 16–18% at this temperature. Finally, 53–55% of the compounds decomposed in the temperature range of 400–550 °C.

##### 3.1.6.5 TGA analysis of Pd@biochar/g-C_3_N_4_-PEI

Qualitative analysis of organic groups on the biochar surface was performed using TGA at a heating rate of 10 °C min^−1^ from 25 to 800 °C.^42^ The TGA curve for Pd@biochar/g-C_3_N_4_-PEI is shown in Fig. 7, diagram e. The initial weight loss of about 2–3% below 150 °C indicates moisture removal. The DTG peak at 115.63 °C confirms this dehydration process. Between 150 and 300 °C, a gradual weight loss (about 13%) occurs due to the degradation of the PEI chain and the decomposition of the surface functional groups.^43^ The main decomposition stage (300 to 800 °C) shows a 12.5% weight loss. This significant decrease indicates the complete degradation of PEI, TDI, g-C_3_N_4_, and organic biochar components.^44^

#### 3.1.7. EDS/mapping analysis

**Fig. 8:**
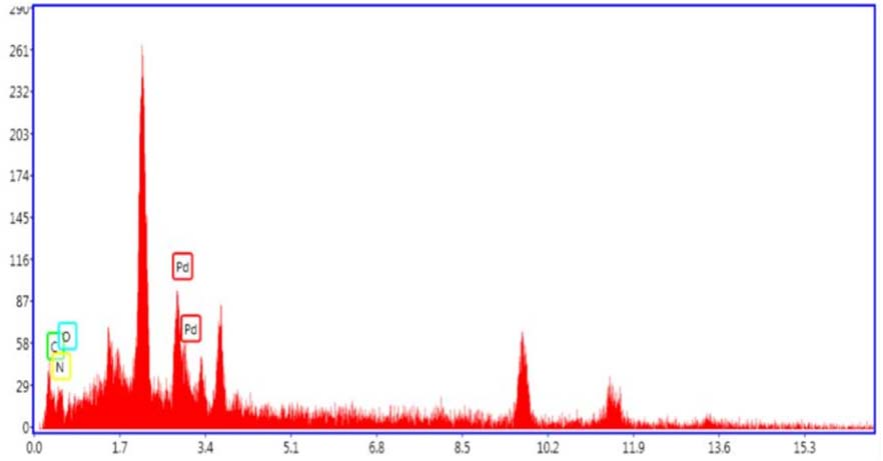
EDX spectrum of Pd@biochar/g-C_3_N_4_-PEI.

**Fig. 9:**
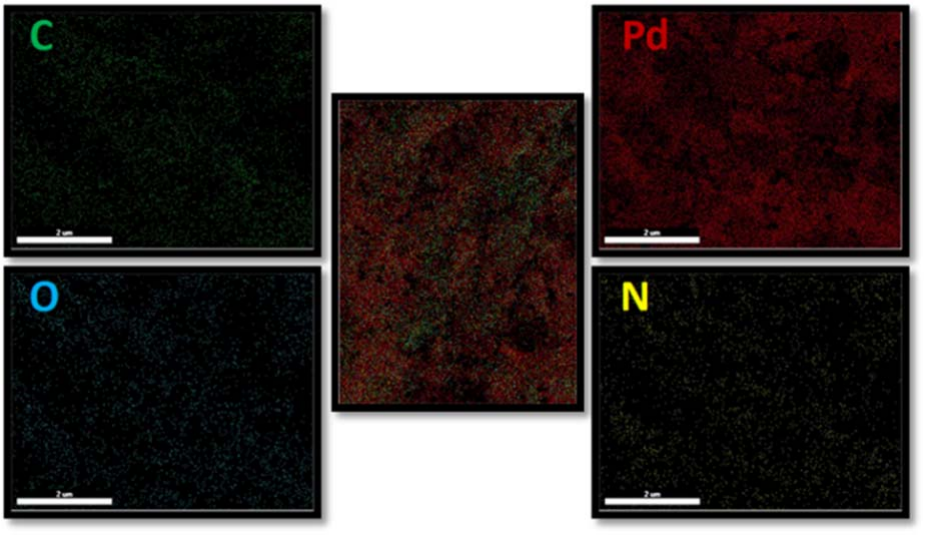
Elemental mapping of carbon, nitrogen, oxygen, and palladium in Pd@biochar/g-C_3_N_4_-PEI.

EDS analysis of Pd@g-C_3_N_4_/biochar-PEI showed the presence of C, O, N, and Pd elements in the catalyst structure, as presented in Fig. 8. WDX analysis also confirmed these findings and evaluated the distribution of elements on the catalyst surface. The mapping results indicate that all elements are evenly distributed throughout the composite, demonstrating the uniform composition of the Pd@g-C_3_N_4_/biochar-PEI catalyst, as shown in Fig. 9. The palladium content was determined to be 2.147 × 10^−3^ mol g^−1^ using ICP-OES, confirming the successful loading of the metal onto the biochar support.

### 3.2 Catalytic activity of Pd@biochar/g-C_3_N_4_-PEI in C–C coupling reactions

After characterization of the catalyst, Pd@biochar/g-C_3_N_4_-PEI was first used in the Stille coupling between iodobenzene and triphenyltin chloride to find the best reaction conditions (Table 1).

**Table 1:** Optimizing reaction conditions for the Stille reaction in the presence of Pd@biochar/g-C_3_N_4_-PEI^a^

Entry	Catalyst (g)	Solvent	Base	Temperature (°C)	Time (min)	Yield%
1	0.001	PEG	K_2_CO_3_	80	100	68
2	0.002	PEG	K_2_CO_3_	80	85	75
3	0.003	PEG	K_2_CO_3_	80	60	86
4	0.004	PEG	K_2_CO_3_	80	25	96
5	0.004	PEG	KOH	80	75	65
6	0.004	PEG	Et_3_N	80	90	68
7	—	PEG	K_2_CO_3_	80	360	—
8	0.004	Water	K_2_CO_3_	Reflux	180	55
9	0.004	Ethyl acetate	K_2_CO_3_	Reflux	220	40
10	0.004	Acetonitrile	K_2_CO_3_	Reflux	170	55
11	0.004	DMF	K_2_CO_3_	Reflux	140	63
12	0.004	*n*-Hexane	K_2_CO_3_	Reflux	240	Trace
13	0.004	Ethanol	K_2_CO_3_	Reflux	140	70
14	0.004	Toluene	K_2_CO_3_	Reflux	240	Trace
15	0.004	PEG	K_2_CO_3_	25	220	30
16	0.004	PEG	K_2_CO_3_	40	110	25
17	0.004	PEG	K_2_CO_3_	50	90	48
18	0.004	PEG	K_2_CO_3_	60	70	68

a Reaction conditions: iodobenzene (1 mmol), Sn(C_6_H_5_)_3_Cl (1 mmol), K_2_CO_3_ (3 mmol), and PEG (3 mL).

First, the amount of catalyst was investigated. Other variables were kept constant: K_2_CO_3_ as the base, PEG-400 as the solvent, and a temperature of 80 °C. As the amount of catalyst increased from 0.001 g to 0.004 g, the yield gradually improved from 68% to 96%. A run without the catalyst (Table 1, entry 7) did not produce any product even after 6 h, confirming that the Pd@biochar/g-C_3_N_4_-PEI catalyst is essential for this reaction. The choice of base made a big difference in the results. Of all the bases tested, K_2_CO_3_ gave the best yield (96%) in just 25 minutes, while the other bases resulted in much lower conversions. Various solvents were also investigated, with PEG-400 performing better than water, ethanol, DMF or *n*-hexane. Non-polar solvents such as *n*-hexane and toluene gave very poor results, probably because the reactants and base did not dissolve well in them. Temperature was critical for the reaction efficiency. When the temperature was reduced from 80 °C to 25 °C, the yield dropped dramatically from 96% to 30%. This suggests that higher temperatures are required for efficient catalyst conversion. From all these optimization experiments, it was found that the best conditions were: 0.004 g of Pd@biochar/g-C_3_N_4_-PEI catalyst, K_2_CO_3_ as the base, PEG-400 as the solvent and a temperature of 80 °C. These conditions gave us a product with a yield of 96% in 25 min (Table 1, entry 4).

**Scheme 1:**
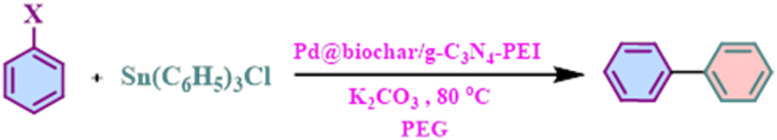
Carbon–carbon coupling reaction of aryl halides with Sn(C_6_H_5_)_3_Cl in the presence of Pd@biochar/g-C_3_N_4_-PEI.

After optimizing the reaction conditions, the Pd@biochar/g-C_3_N_4_-PEI catalyst was tested with various aryl halides in the acetyl coupling reaction using triphenyltin chloride (Table 2 and Scheme 1). The catalyst performed very well on various substrates and achieved high TON and TOF values in relatively short times. A clear pattern was observed: the reaction rates of aryl iodides did not differ significantly from those of aryl bromides. For example, iodobenzene (entry 1) was converted in 96% yield with a TOF of 266 h^−1^. In comparison, bromobenzene (entry 2) had a slightly lower TOF of 209 h^−1^ with a yield of 90%. This trend makes sense when considering that the carbon–iodine bond is weaker and more easily undergoes oxidative addition to palladium. Substrates with electron-donating and electron-withdrawing groups did not differ much in reaction rates and times and performed almost similarly. The best example was 4-iodotoluene (entry 4), which showed a TOF of 266 h^−1^ with a yield of 96%. 4-Iodoanisole (entry 6) also performed well, giving a yield of 92% in 30 min with a TOF of 213 h^−1^. On the other hand, 4-bromonitrobenzene (entry 5) with a TOF of 211 h^−1^ reached a yield of 91%. Similarly, 4-bromobenzaldehyde (entry 8) was obtained in 75% yield with a TOF of 87 h^−1^. 1-Chloro-4-iodobenzene (entry 3) underwent selective monocoupling in 95% yield in 35 min. The catalyst indeed acted selectively and among the three possible products, chlorobiphenyl was synthesized in 95% yield, consistent with GC-mass analysis (SI). Even 2-iodobenzoic acid (entry 10), which has a bulky carboxylic acid group right next to the iodine, still achieved 93% yield in 30 min. The catalyst also worked with heteroaromatic compounds – 2-bromophthalene (entry 9) with a TOF of 184 h^−1^ in 35 min – and achieved 92% yield. In summary, the Pd@biochar/g-C_3_N_4_-PEI catalyst showed a good substrate range in the Stille coupling reaction. The TON values ranged from 87 to 266, indicating strong catalytic activity.

**Table 2:** Stille C–C coupling reaction for synthesis of biphenyl derivatives catalyzed by Pd@biochar/g-C_3_N_4_-PEI^a^

Entry	Aryl halide	Time (min)	Yield%	TON	TOFh^−1^	M. P. (°C)	Ref.
1	Iodobenzene	25	96	111	266	66–68	68 (ref. 45)
2	Bromobenzene	30	90	104	209	65–67	66–68 (ref. 45)
3	1-Chloro-4-iodobenzen	35	95	110	190	71–74	72–73 (ref. 46)
4	4-Iodotoluene	25	96	111	266	44–47	44–47 (ref. 47)
5	4-Bromonitrobenzene	30	91	105	211	111–114	113–115 (ref. 48)
6	4-Iodoanisole	30	92	106	213	82–84	82–84 (ref. 49)
7	2-Iodoanisole	35	89	103	178	(Oil)	Oil^50^
8	4-Bromobenzaldehyde	60	75	87	87	54–56	55–57 (ref. 51)
9	2-Bromonaphthalene	35	92	106	184	103–105	104–106 (ref. 51)
10	2-Iodobenzoic acid	30	93	108	216	110–112	110–112 (ref. 19)

a Reaction conditions: aryl halide (1 mmol), Sn(C_6_H_5_)_3_Cl (1 mmol), K_2_CO_3_ (3 mmol), Pd@biochar/g-C_3_N_4_-PEI (4 mg), and PEG (3 mL) at 80 °C.

**Scheme 2:**
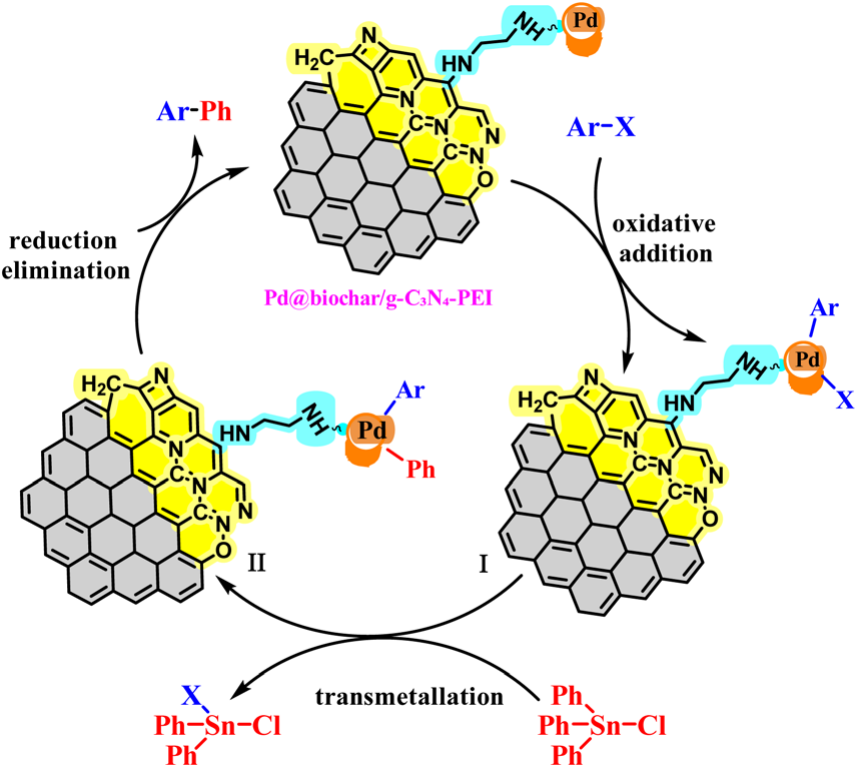
Proposed mechanism for the Stille cross-coupling reaction in the presence of Pd@biochar/g-C_3_N_4_-PEI.

Scheme 2 outlines the proposed mechanism for the Pd-catalyzed Stille coupling reaction. The process begins with oxidative addition of the aryl halide to generate intermediate I. Transmetalation between this intermediate and triphenyltin chloride produces intermediate II, which subsequently undergoes reductive elimination to afford the desired product while simultaneously regenerating the palladium catalyst.^52^

### 3.3 Catalytic activity of Pd@biochar/g-C_3_N_4_-PEI in C–N coupling reactions

**Scheme 3:**
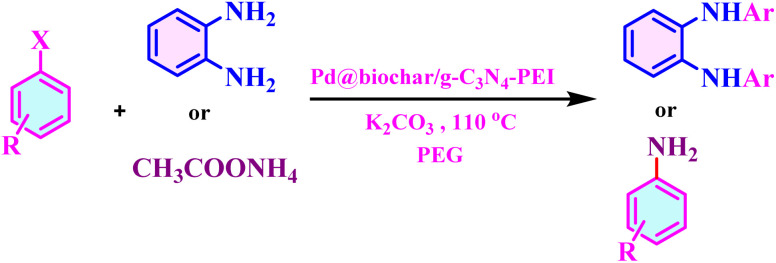
C–N coupling reaction of *O*-phenylenediamine or ammonium acetate with aryl halides in the presence of Pd@biochar/g-C_3_N_4_/-PEI.

C–N coupling reactions were also investigated using the Pd@biochar/g-C_3_N_4_-PEI nanocomposite. The reaction between iodobenzene and ammonium acetate was used as a model reaction (Scheme 3). Various conditions, including catalyst amount, solvent, base, and temperature, were thoroughly investigated (Table 3). The effect of catalyst loading was investigated while keeping the other variables (110 °C, K_2_CO_3_, and PEG-400) constant. Increasing the catalyst loading from 0.001 g to 0.006 g led to an improvement in the yield from 67% to 95%. Further increasing the catalyst loading beyond 0.006 g did not increase the yield. Accordingly, 0.006 g was selected as the optimal catalyst loading (Table 3, entry 6). The reaction without the catalyst also did not produce any product. Among the solvents used, PEG-400 had the highest efficiency (95%). Polar aprotic solvents, such as DMF and acetonitrile, had moderate yields.

**Table 3:** Optimizing reaction conditions for C–N coupling reactions in the presence of Pd@biochar/g-C_3_N_4_-PEI^a^

Entry	Catalyst (g)	Solvent	Base	Temperature (°C)	Time (min)	Yield (%)
1	0.001	PEG	K_2_CO_3_	110	65	67
2	0.002	PEG	K_2_CO_3_	110	55	75
3	0.003	PEG	K_2_CO_3_	110	50	82
4	0.004	PEG	K_2_CO_3_	110	45	88
5	0.005	PEG	K_2_CO_3_	110	42	92
6	0.006	PEG	K_2_CO_3_	110	40	95
7	—	PEG	K_2_CO_3_	110	360	N.R.
8	0.006	Water	K_2_CO_3_	Reflux	200	60
9	0.006	DMF	K_2_CO_3_	Reflux	140	66
10	0.006	Ethanol	K_2_CO_3_	Reflux	150	70
11	0.006	Ethyl acetate	K_2_CO_3_	Reflux	240	30
12	0.006	*n*-Hexane	K_2_CO_3_	Reflux	360	Trace
13	0.006	Acetonitrile	K_2_CO_3_	Reflux	170	62
14	0.006	Toluene	K_2_CO_3_	Reflux	220	50
15	0.006	PEG	KOH	110	140	60
16	0.006	PEG	Et_3_N	110	160	68
17	0.006	PEG	Na_2_CO_3_	110	150	78
18	0.006	PEG	K_2_CO_3_	60	160	52
19	0.006	PEG	K_2_CO_3_	80	120	68
20	0.006	PEG	K_2_CO_3_	90	90	80

a Reaction conditions: iodobenzene (1 mmol), ammonium acetate (1 mmol) or *o*-phenylenediamine (0.5 mmol), and PEG (3 mL).

The use of a base also significantly affected the reaction outcome. Therefore, different bases were used, with K_2_CO_3_ providing the best results in terms of yield and reaction time. At 110 °C, the reaction yield was 95%; when the temperature reached 60 °C, the yield became 52%. Therefore, the optimal reaction conditions were as follows: 0.006 g Pd@biochar/g-C_3_N_4_-PEI catalyst, K_2_CO_3_ as the base, PEG-400 as the solvent, and a temperature of 110 °C. Under these conditions, the product was obtained in 95% yield within 40 min (Table 3, entry 6).

Using the optimized conditions, the Pd@biochar/g-C_3_N_4_-PEI catalyst was investigated with various aryl halides and ammonium acetate or *o*-phenylenediamine (Table 4). With iodobenzene and ammonium acetate, the yield was 95% (entry 1) in 40 min, while bromobenzene (entry 5) required 60 min to reach 88% yield. Electron-donating substituents performed well; 4-iodoanisole (entry 3) gave 86% yield in 50 min. When *o*-phenylenediamine was used as another amine, a longer reaction time was generally required due to its bulkier structure. Iodobenzene (entry 6) gave 92% yield in 55 min (TOF = 77 h^−1^), while bromobenzene (entry 7) required 85 min to reach 86% yield (TOF = 47 h^−1^). The *o*-phenylenediamine-substituted aryl iodides showed good results: 4-iodotoluene (entry 10) gave 91% yield in 60 min with a TOF of 70 h^−1^, while the methoxy-substituted compounds (entries 9 and 10) required 70–75 min to reach 84–88% yield.

**Table 4:** C–N coupling reaction for synthesis of aryl amine derivatives catalyzed by Pd@biochar/g-C_3_N_4_-PEI^a^

Entry	Aryl halide	Amine	Time (min)	Yield%	TON	TOF h^−1^	M. P. (°C)	Ref.
1	Iodobenzene	Ammonium acetate	40	95	73	110	Oil	53
2	Chlorobenzene	Ammonium acetate	70	85	65	56	Oil	53
3	4-Iodoanisole	Ammonium acetate	50	90	69	83	57–58	57–59 (ref. 54)
4	1-Iodo-4-nitrobenzene	Ammonium acetate	55	89	68	75	145–147	147–149 (ref. 55)
5	Bromobenzene	Ammonium acetate	60	88	68	68	Oil	53
6	Iodobenzene	*O*-phenylenediamine	55	92	71	77	108–110	105–107 (ref. 56)
7	Bromobenzene	*O*-phenylenediamine	85	86	66	47	108–110	107–108 (ref. 57)
8	4-Iodotoluene	*O*-phenylenediamine	60	91	70	70	44–47	35–36 (ref. 57)
9	4-Iodoanisole	*O*-phenylenediamine	70	84	65	55	103–105	102–104 (ref. 19)

a Reaction conditions: aryl halide (1 mmol), ammonium acetate (1 mmol) or *o*-phenylenediamine (0.5 mmol), K_2_CO_3_ (3 mmol), Pd@biochar/g-C_3_N_4_ (6 mg), and PEG (3 mL) at 110 °C.

The TON values ranged from 47 to 110, indicating efficient catalytic turnover. The catalyst showed a wide substrate range and good tolerance to different functional groups.

**Scheme 4:**
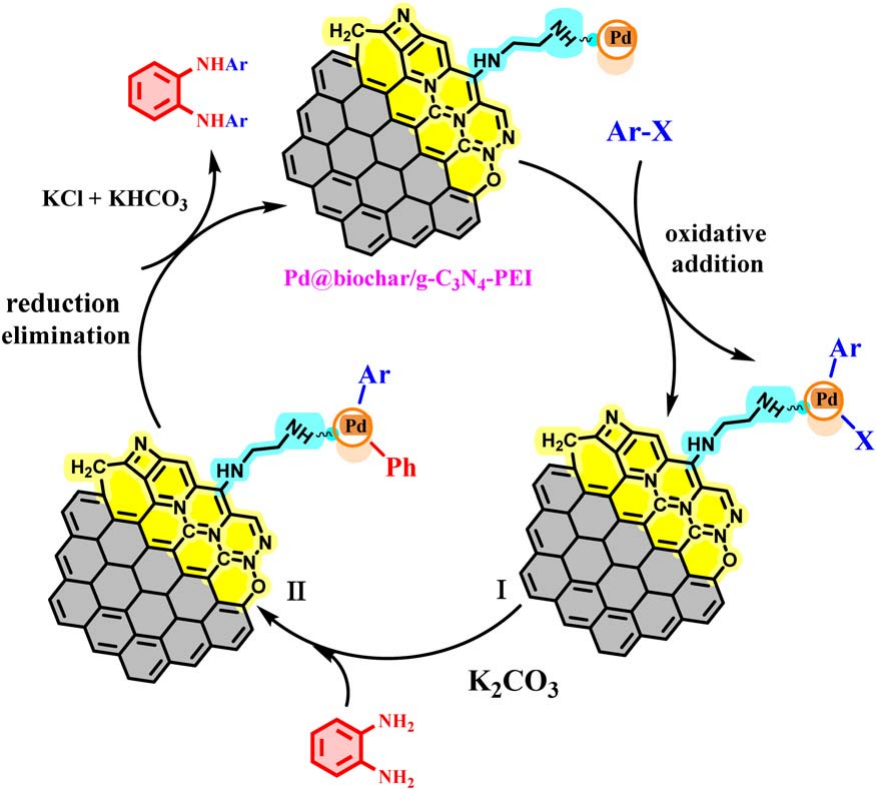
Proposed mechanism for the Pd@biochar/g-C_3_N_4_-PEI catalyzed C–N coupling.

Scheme 4 shows the proposed mechanism for the Pd@biochar/g-C_3_N_4_-PEI catalyzed C–N coupling. The cycle starts with oxidative addition of the aryl halide to Pd(0), forming a Pd(ii)–aryl intermediate (I). The weaker C–I bond explains why aryl iodides react faster than bromides in our experiments. Next, the amine coordinates to palladium and gets deprotonated by the base, giving intermediate II. Finally, reductive elimination forms the C–N bond and regenerates the Pd(0) catalyst. The high temperature (110 °C) we found optimal likely helps this final step proceed efficiently. The biochar/g-C_3_N_4_ support stabilizes the palladium and maintains catalytic activity.^58^

The performance of the Pd@biochar/g-C_3_N_4_-PEI catalyst was investigated in comparison with previously reported catalysts. The synthesized catalytic system is able to achieve 96% efficiency under milder conditions (80 °C, 22 min), while many Pd and Cu catalysts require longer reaction times and higher temperatures to achieve similar efficiency. This better activity can be attributed to two key factors: first, the homogeneous and optimal dispersion of palladium nanoparticles, and second, the presence of active functional groups on the biochar support that provide an effective interaction to enhance stability and improve catalytic activity (Table 5).

**Table 5:** Comparison results for the catalytic activity of Pd@biochar/g-C_3_N_4_-PEI with other catalysts in C–C and C–Ncoupling reactions

Entry	Conditions	Time	Yield (%)	Ref.
1^a^	Aryl chloride (1.0 mmol), organostannane (1.1 mmol), CsF (2.0 mmol), 50 °C, Pd (0.5 mol%), and THF	3 h	93	59
2^a^	3 mmol of aryl halide, 3 mmol of tributylphenyltin, 9 mmol of LiCl, and 0.002 g of IRMOF-3-PI-Pd catalyst (Pd content: 2.40 × 10^−2^ mol%) in 5 mL of ethanol at 80 °C	2 h	90	60
3^a^	1 mmol of aryl halide, 1 mmol of tributylphenyltin, 2.89 mmol of LiCl, and 0.05 g of Pd(0)-MCM-41in 4 mL of DMF at 100 °C	20 h	72	61
4^a^	Pd@biochar/g-C_3_N_4_/-PEI (4 mg), PEG, K_2_CO_3_, 80 °C	20 min	96	This work
5^b^	Cu_2_O (5 mol%), NH_3_ (10 equiv.), H_2_O–NMP (1 : 1), MW, 110 °C	10–12 h	79–93	62
6^b^	0.2 mmol aryl halide, 2 mol% Pd(OAc)_2_, 8 mol% ligand, 2 equiv. NaOtBu, 2.0 mL of 0.5 M NH_3_/1,4-dioxane, 10 bar N_2_	24 h	80	63
7^b^	Pd@biochar/g-C_3_N_4_-PEI (6 mg), PEG, K_2_CO_3_, 110 °C	40 min	95	This work

a Different catalysts for the C–C Stille coupling reaction of iodobenzene and Sn(C_6_H_5_)_3_Cl.

b Different catalysts for the C–N coupling reaction of 2-bromonaphthalene and ammonia.

#### 3.3.1. Hot filtration test and recovery of the catalyst

Hot filtration confirmed the heterogeneous nature of Pd@biochar/g-C_3_N_4_-PEI. The catalyst yielded 58% biphenyl in the coupling reaction of iodobenzene and Sn(C_6_H_5_)_3_Cl after 13 minutes. Further validation involved removing the catalyst halfway through a replicated reaction; the subsequent completion of the reaction without the catalyst resulted in 61% biphenyl, confirming negligible palladium leaching. The robust Pd@biochar/g-C_3_N_4_-PEI catalyst exhibits excellent stability and reusability in Stille cross-coupling reactions. Its high conversion rate and minimal palladium leaching make it a promising sustainable catalysis candidate, potentially reducing reliance on homogeneous palladium catalysts and mitigating environmental metal contamination.

**Fig. 10:**
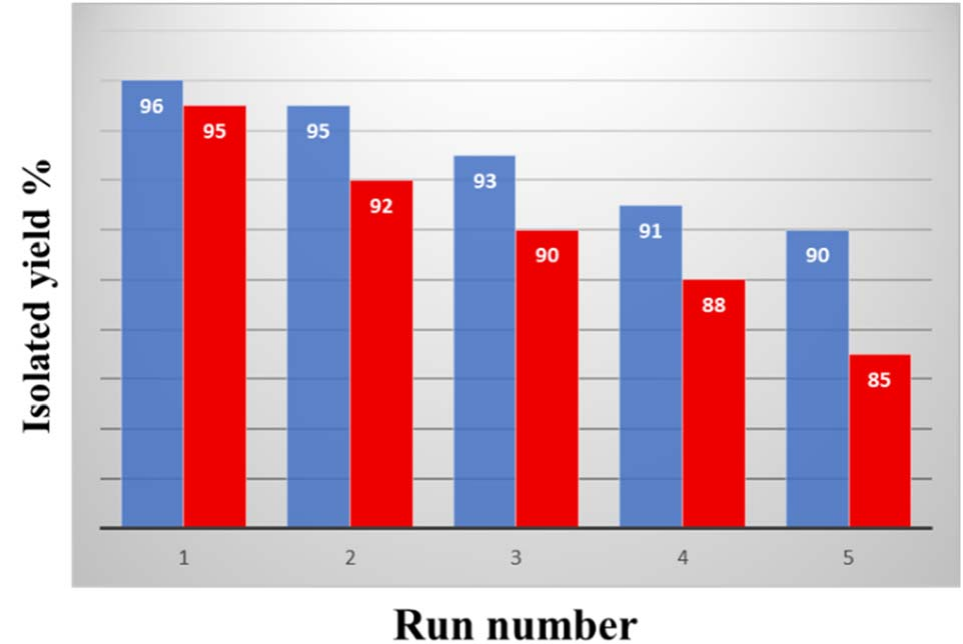
Recyclability study of Pd@biochar/g-C_3_N_4_-PEI as a catalyst in C–C (blue column) and C–N (red column) coupling reactions.

**Fig. 11:**
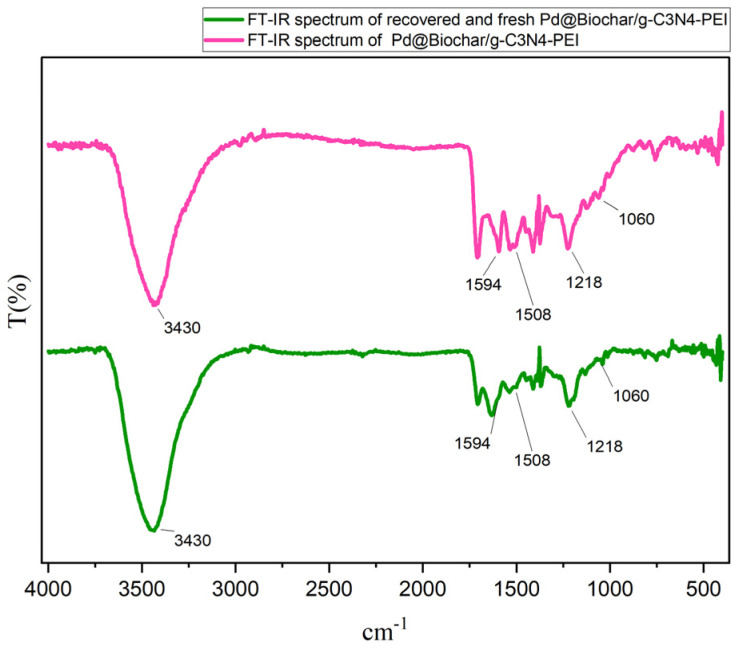
FT-IR spectrum of recovered and fresh Pd@biochar/g-C_3_N_4_-PEI.

**Fig. 12:**
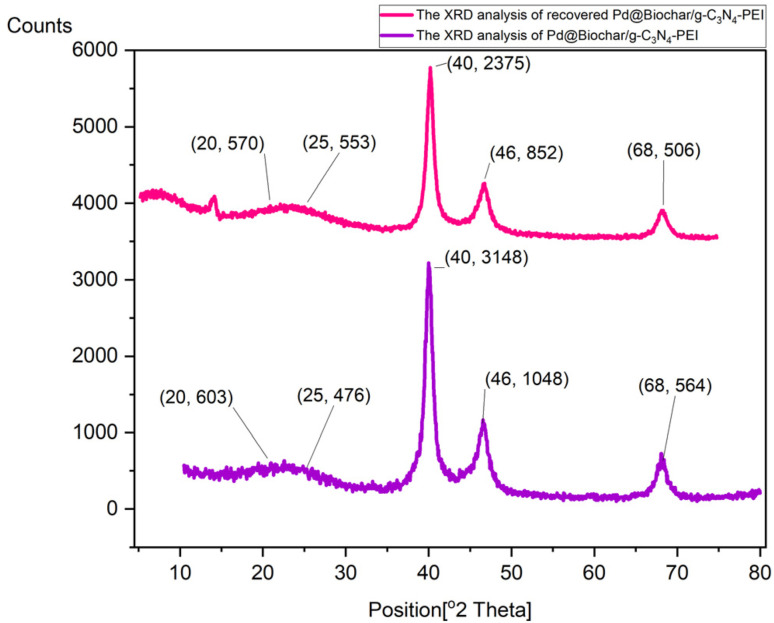
The XRD analysis of fresh and recovered Pd@biochar/g-C_3_N_4_-PEI.

The recoverability of a catalyst is critical for assessing its practical applications. The heterogeneous nature of the Pd@biochar/g-C_3_N_4_-PEI catalyst allows for its recovery and reuse. Therefore, its reusability was evaluated in the synthesis of aryl amines and biphenyls, using the C–C cross-coupling of iodobenzene with Sn(C_6_H_5_)_3_Cl and the C–N cross-coupling of iodobenzene with ammonium acetate as model reactions. These reactions were initially performed under optimized conditions (Table 1, entry 4 and Table 3, entry 6). After each reaction, the catalyst was recovered and its catalytic activity was re-evaluated in subsequent reactions. The results (Fig. 10) demonstrate that the catalyst can be reused for at least 5 consecutive runs in both C–C and C–N coupling reactions. The slight activity decrease after each run may be due to catalyst loss during recovery or Pd nanoparticle leaching from the biochar/g-C_3_N_4_/PEI support. The catalyst retains significant activity, suggesting its potential for sustainable cross-coupling applications. FT-IR spectra and XRD analysis confirm the stability of the recovered Pd@biochar/g-C_3_N_4_-PEI catalyst under reaction conditions (Fig. 11 and 12). The nearly identical FT-IR spectra and XRD analysis before and after the reaction indicate that the catalyst's structure and composition remained unchanged. The consistent FT-IR spectra and unaltered XRD patterns indicate high catalyst stability and reusability. The preservation of surface functional groups and the crystalline structure confirm the catalyst's resistance to degradation, ensuring long-term viability and cost-effectiveness for industrial applications.

## 4 Conclusion

In this study, a Pd@biochar/g-C_3_N_4_-PEI heterogeneous catalyst was synthesized. Initially, biochar was produced from pyrolyzing walnut shells. Next, thermal polycondensation was employed to prepare biochar/g-C_3_N_4_ composites and toluene diisocyanate was used to covalently bond PEI to the biochar/g-C_3_N_4_ surface. Subsequently, the Pd–PEI complex was supported on the surface of the prepared biochar, directly. This catalyst was characterized using FT-IR, BET, SEM, TEM, TGA, ICP, EDS, and XRD techniques. The Pd@biochar/g-C_3_N_4_-PEI catalyst effectively facilitated the Stille and C–N coupling reactions in the green solvent polyethylene glycol, enabling the synthesis of biphenyls and aryl amines. The reaction parameters were optimized by evaluating the catalyst efficiency under varying temperatures, reaction times, and catalyst loadings. The method's effectiveness across diverse substrates was demonstrated in the Stille and C–N coupling reactions using aryl halides with both electron-donating and electron-withdrawing groups. The Pd@biochar/g-C_3_N_4_-PEI catalyst enabled the synthesis of products in moderate to excellent yields, demonstrating its potential in organic chemistry. The Pd@biochar/g-C_3_N_4_/-PEI catalyst proved recyclable, retaining activity over several uses, which underscores its economic and environmental advantages for sustainable different applications by reducing waste and minimizing the environmental impact of catalyst production and disposal.

## Author contributions

Maryam Nouri: methodology, validation, and investigation. Maryam Hajjami: supervision, resources, project administration, funding acquisition, and conceptualization. Arash Ghorbani-Choghamarani: supervision and resources. Zahra Siahpour: investigation and writing – review.

## Conflicts of interest

There are no conflicts to declare.

## Supplementary Material

NA-OLF-D6NA00144K-s001

## Data Availability

The datasets used and/or analysed during the current study are available from the corresponding author on reasonable request. Supplementary information (SI) is available. See DOI: https://doi.org/10.1039/d6na00144k.
