# Supplementary material for: Stille- and Ullmann-type coupling reactions catalysed by palladium supported biochar/g-C3N4-polyethyleneimine as a heterogeneous nanocatalyst
Source: Nanoscale Adv. 2026 Jul 13;8(15):4372–84. doi: 10.1039/d6na00144k (PMC13359361; doi:10.1039/d6na00144k)
Supplement: NA-008-D6NA00144K-s001 [file NA-008-D6NA00144K-s001.pdf]

Supplementary Information (SI) for Nanoscale Advances.
This journal is © The Royal Society of Chemistry 2026

# Stille and Ulmann Type Coupling Reactions Catalysed by Palladium Supported Biochar/g-C3N4- polyethyleneimine as Heterogeneous Nanocatalyst

Maryam Nouri, Maryam Hajjami*, Arash Ghorbani-Choghamarani and Zahra Siahpour

| Contents | |
|---|---|
| Figure 1,2 | **1,1'-Biphenyl**: White solide, M.P: 66- 68 $^{O}C$<br>**$^{1}$H NMR** (500 MHz, DMSO) δ 7.68 – 7.60 (m, 2H), 7.46 (t, *J* = 7.7 Hz, 2H), 7.39 – 7.32 (m, 1H).<br>**$^{13}$C NMR** (126 MHz, DMSO) δ 158.9, 139.8, 132.5, 128.8, 127.7, 126.7, 126.1, 114.3, 55.1. |
| Figure 3,4 | **4-methoxy-1,1'-biphenyl:** White solide, M.P: 82-84 $^{O}C$<br>**$^{1}$H NMR** (500 MHz, DMSO) δ 7.64 – 7.54 (m, 4H), 7.45 – 7.38 (m, 2H), 7.32 – 7.25 (m, 1H), 7.04 – 6.96 (m, 2H), 3.78 (s, 3H).<br>**$^{13}$C NMR** (126 MHz, DMSO) δ 158.9, 139.8, 132.5, 128.8, 127.7, 126.7, 126.1, 114.3, 55.1. |
| Figure 5,6 | **4-methyl-1,1'-biphenyl:** White solide, M.P: 44-47 $^{O}C$<br>**$^{1}$H NMR** (250 MHz, DMSO) δ 7.61 (d, *J* = 10.4 Hz, 2H), 7.44 (dd, *J* = 9.1, 2.0 Hz, 1H), 7.24 (d, *J* = 8.2 Hz, 2H), 2.31 (s, 3H).<br>**$^{13}$C NMR** (63 MHz, DMSO) δ 140.5, 137.7, 137.1, 129.9, 129.3, 127.5, 127.3, 126.9, 126.8, 21.0. |
| Figure 7,8 | **Aniline:** Oile<br>**$^{1}$H NMR** (300 MHz, DMSO) δ 7.72 – 7.63 (m, 3H), 7.53 – 7.44 (m, 3H), 7.42 – 7.34 (m, 1H).<br>**$^{13}$C NMR** (75 MHz, DMSO) δ 140.6, 129.4, 127.9, 127.1. |
| Figure 9,10 | **N, N-bis(1,1'byphenyl)-1,2-benzendiamine:** Yellow solide, M.P: 44-47 $^{O}C$<br>**$^{1}$H NMR** (300 MHz, DMSO) δ 7.67 (dd, *J* = 8.1, 1.6 Hz, 3H), 7.13 (dd, *J* = 8.4, 6.9, 1.6 Hz, 3H), 6.84 (dd, *J* = 8.3, 1.4 Hz, 3H), 6.60 (dd, *J* = 8.3, 7.0, 1.4 Hz, 3H), 6.41 (s, 4H).<br>**$^{13}$C NMR** (75 MHz, DMSO) δ 145.8, 140.6, 137.1, 131.7, 122.2, 117.1, 116.0. |

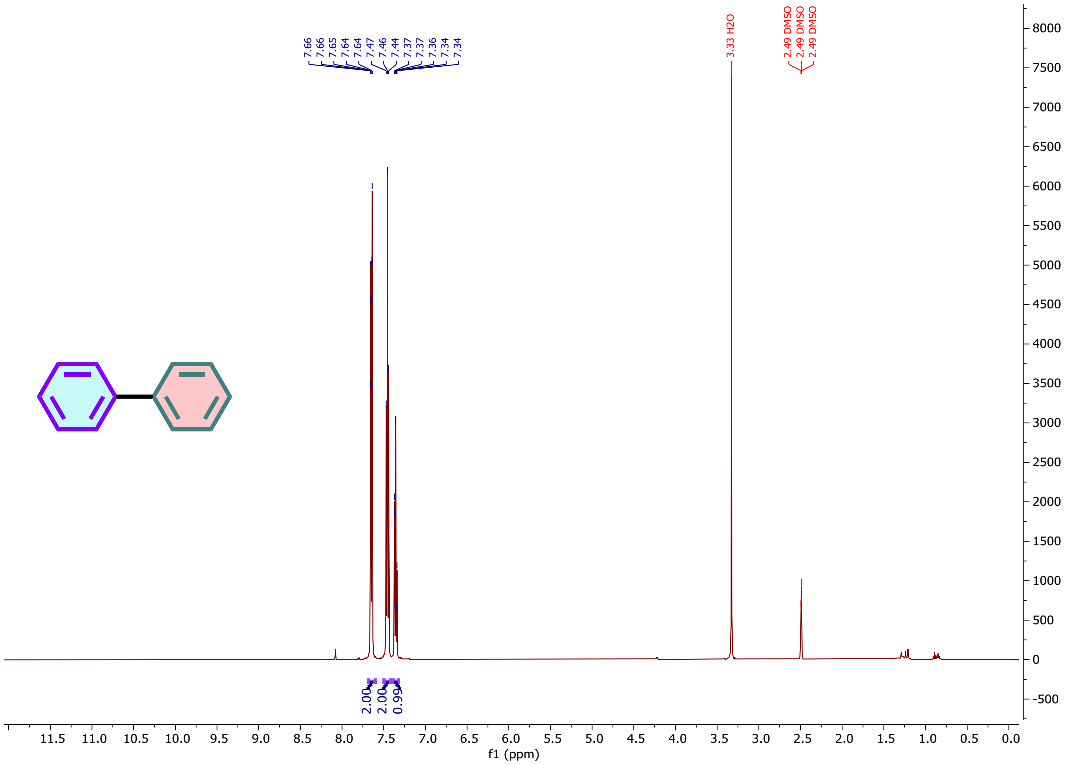


**Figure 1:** $^{1}H$ NMR of 1, 1'byphenyl

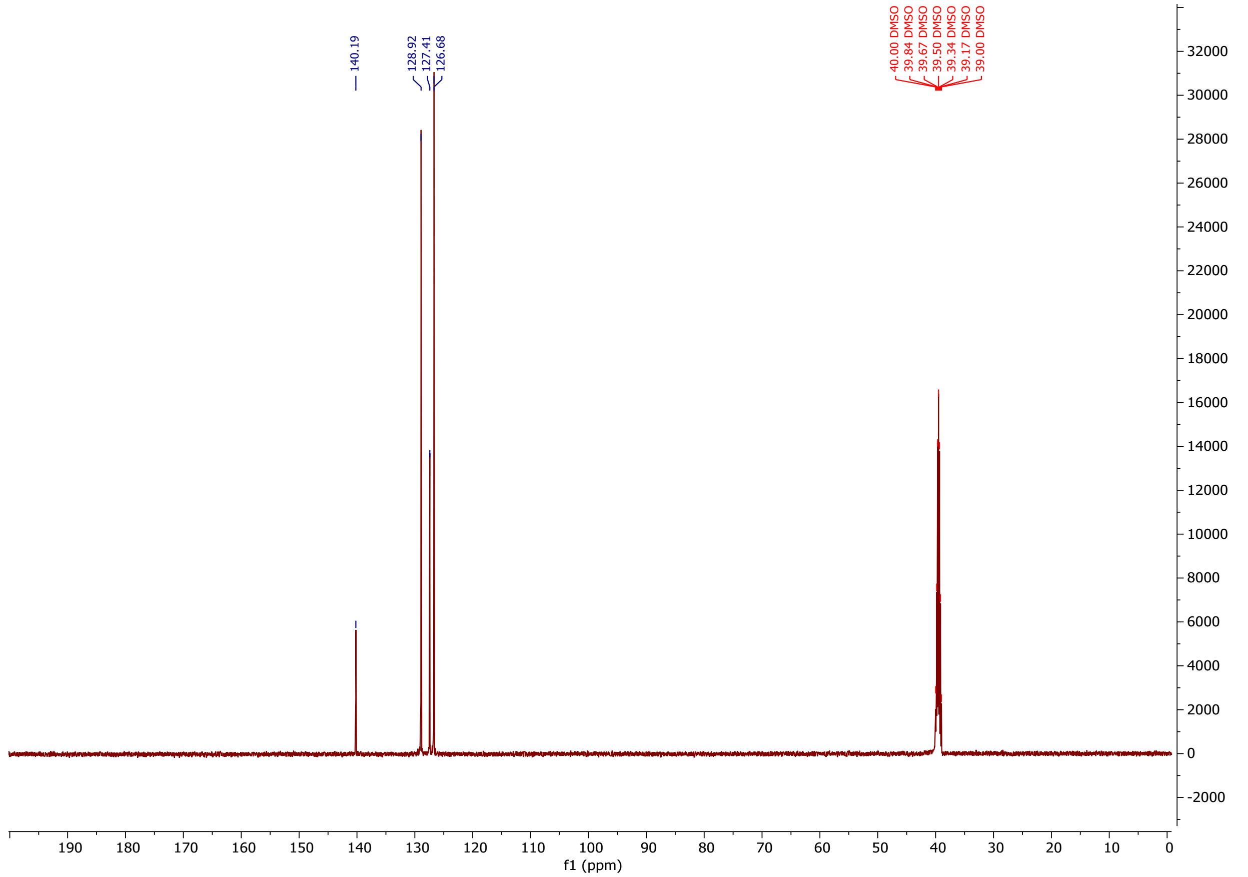


**Figure 2:** $^{13}C$ NMR of 1, 1'byphenyl

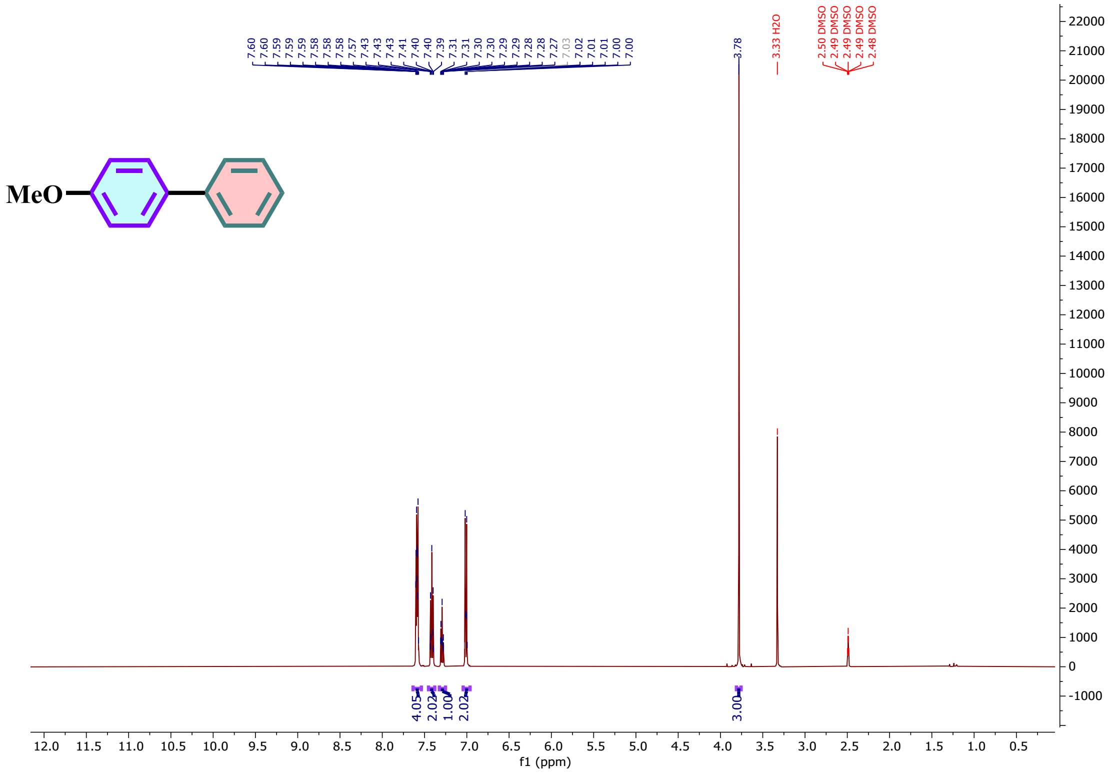


**Figure 3:** $^{1}$H NMR of 4-Methoxy-1, 1'byphenyl

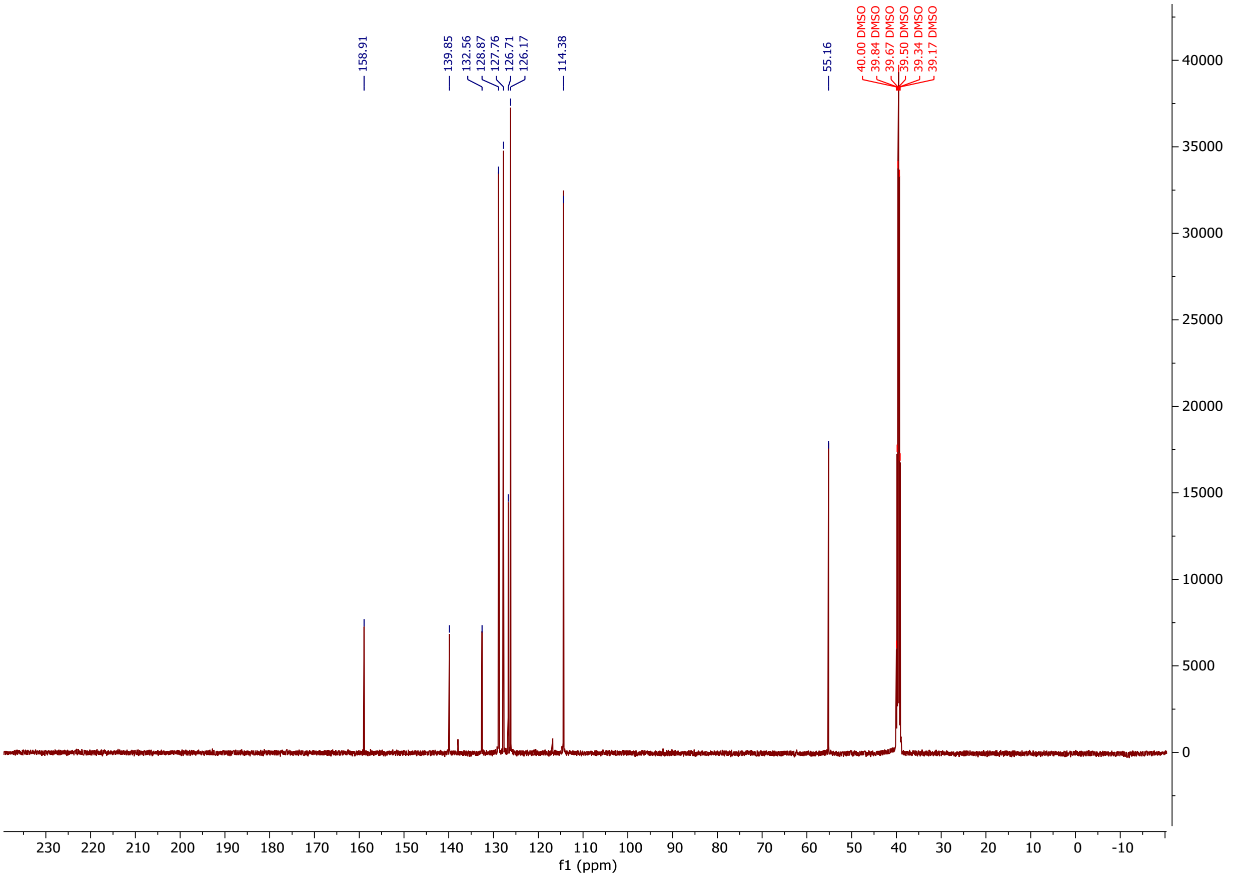


**Figure 4:** $^{13}$C NMR of 4-Methoxy-1, 1'byphenyl

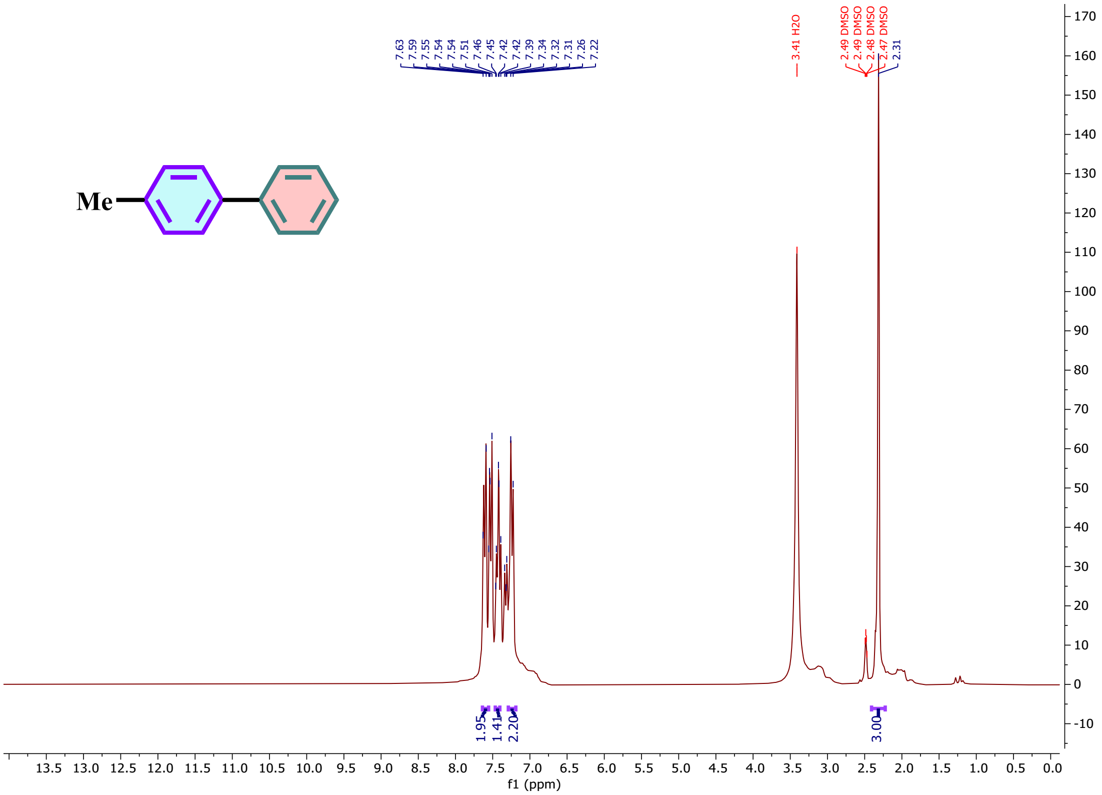

**Figure 5:** $^{1}$H NMR of 4-Methyl-1, 1'byphenyl

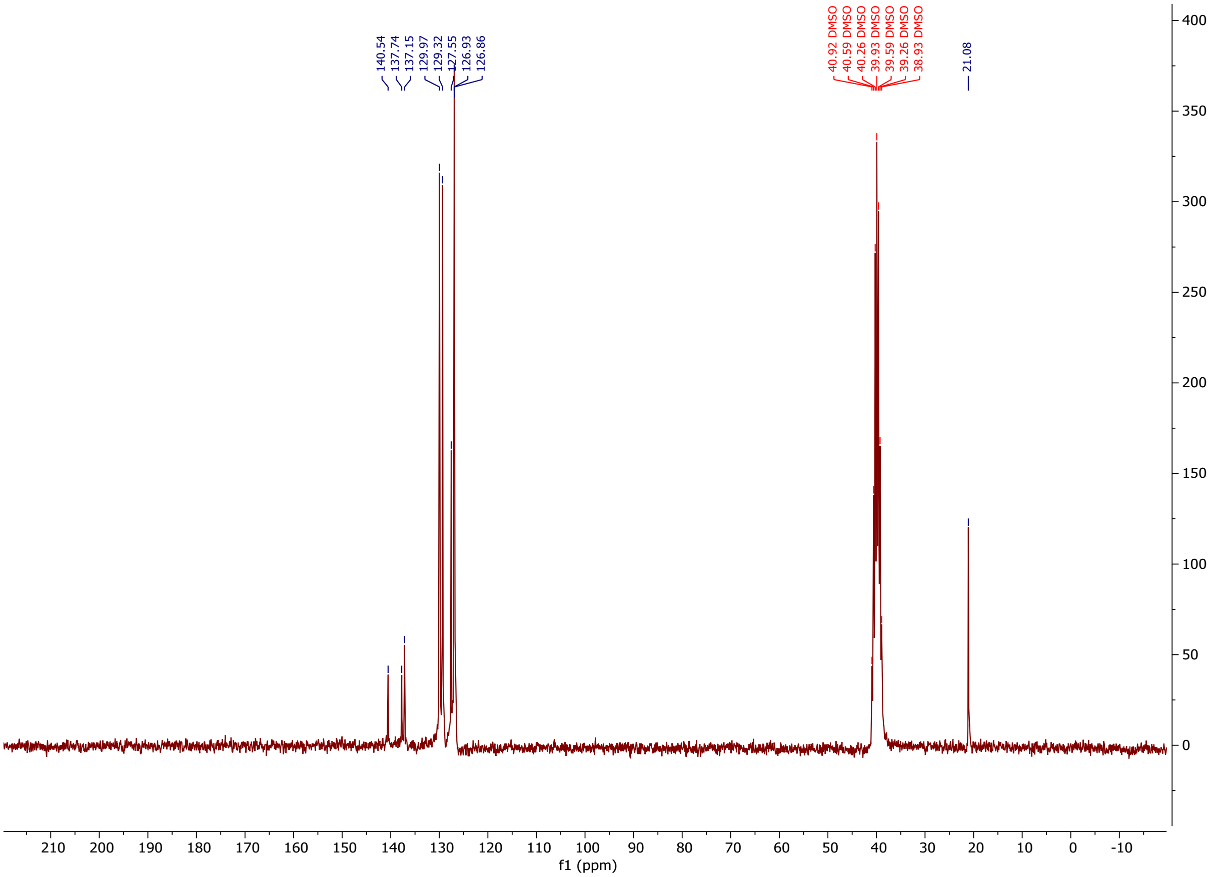


**Figure 6:** $^{13}$C NMR of 4-Methyl-1, 1’byphenyl

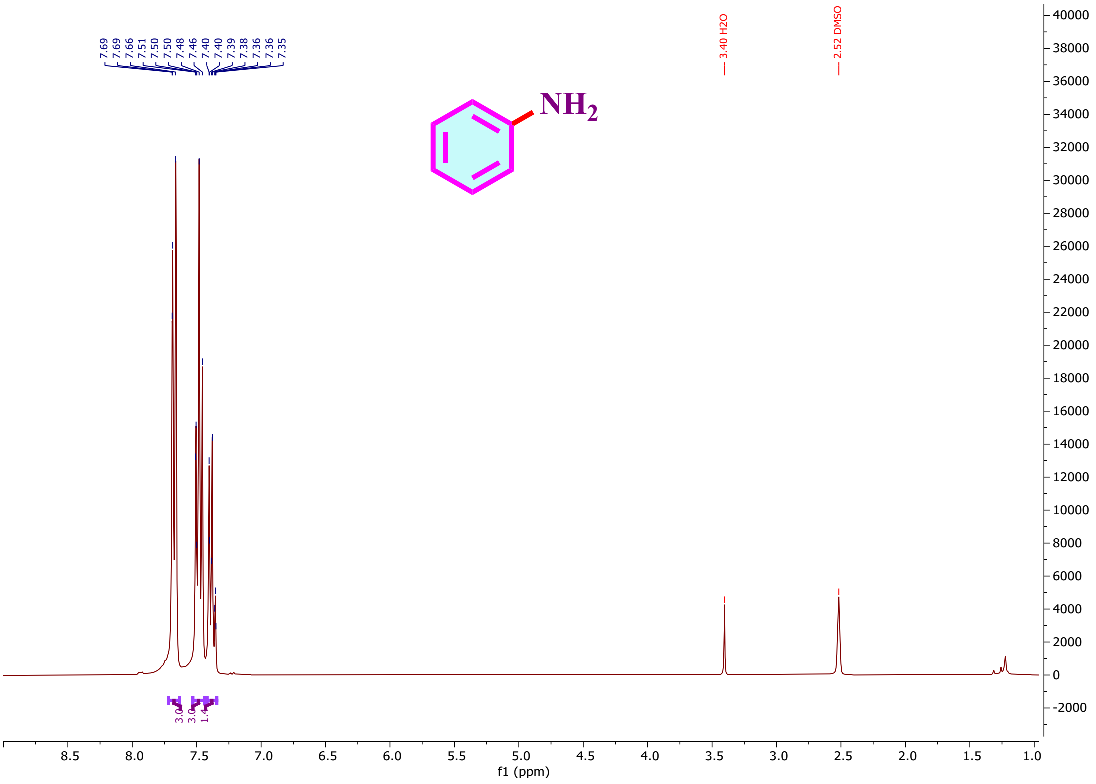


**Figure 7:** $^{1}$H NMR of Aniline

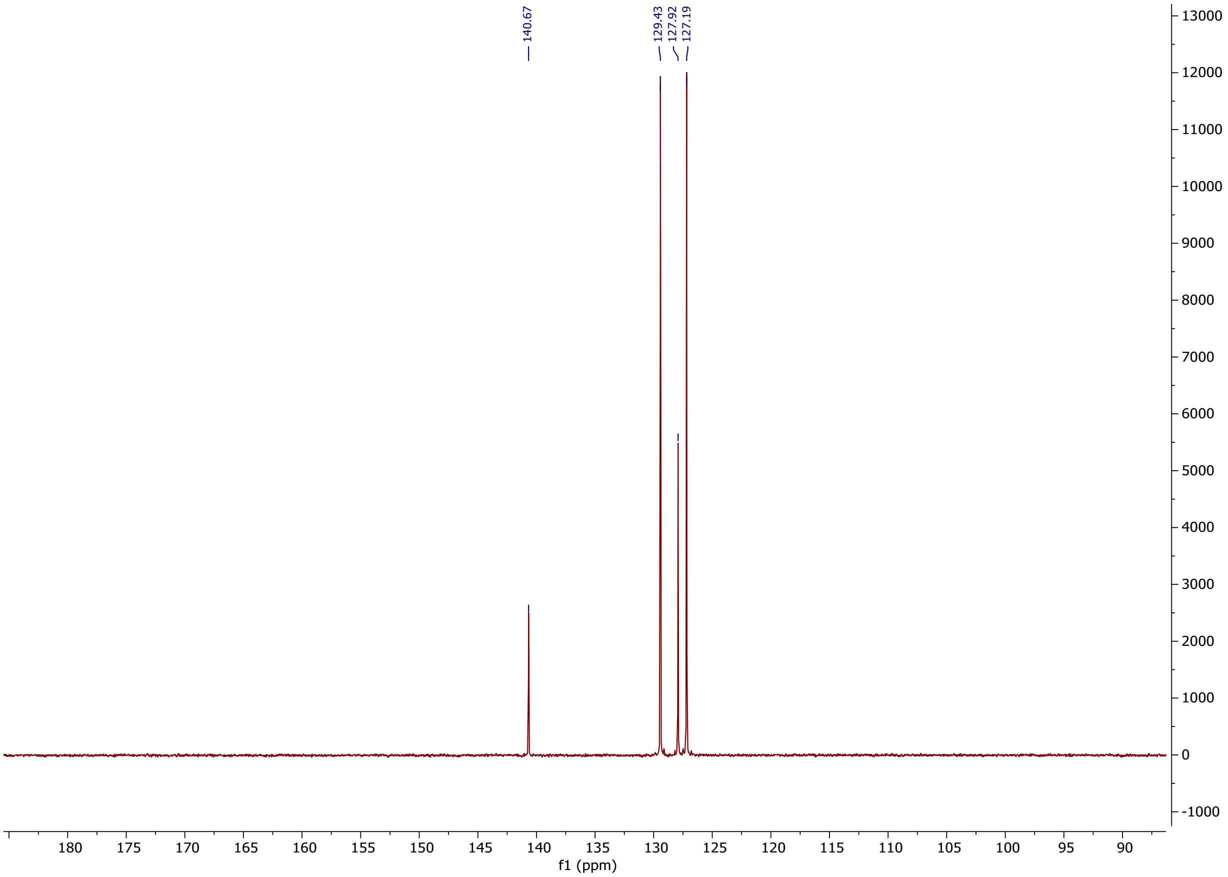


**Figure 8:** $^{13}$C NMR of Aniline

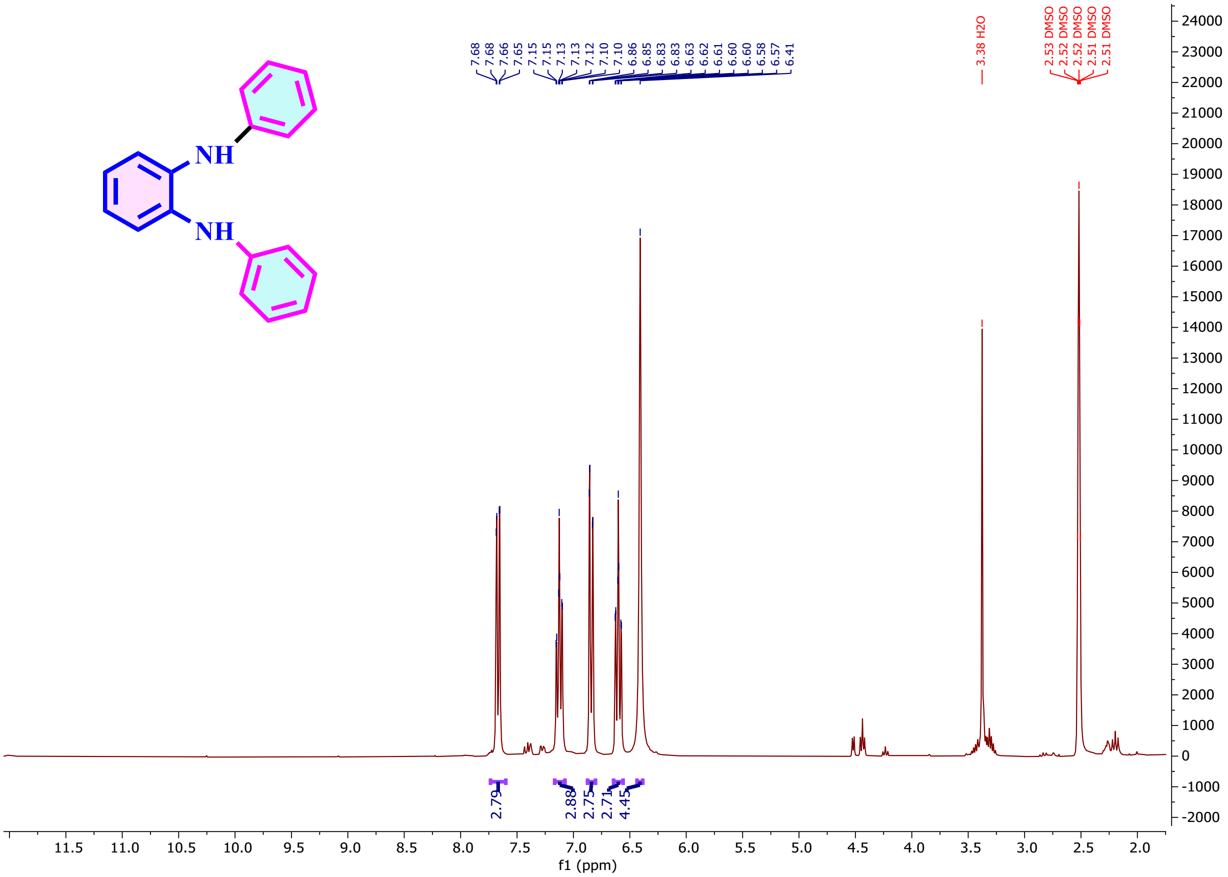

**Figure 9:** $^{1}$H NMR of  N, N-bis(1,1'byphenyl)-1,2-benzendiamine

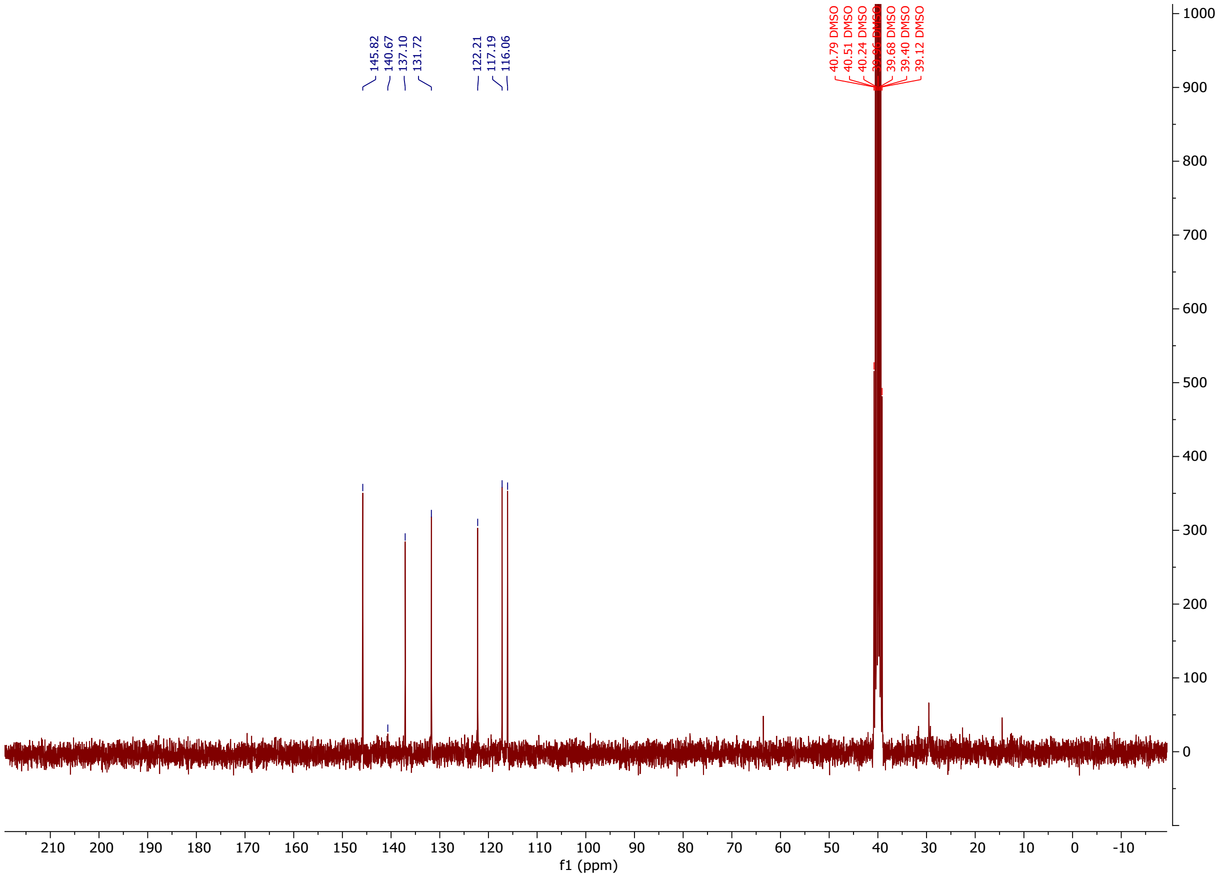


**Figure 10:** $^{13}$C NMR N, N-bis(1,1'byphenyl)-1,2-benzendiamine

- **Gas Chromatograph-Mass spectrometer(GC-MS)**

| | |
|---|---|
| Lock Peak Width: | No |
| Parameters: | Local |
| Peak Width (sec): | 4.0 |
| Slope Sensitivity (SN): | 10 |
| Tangent %: | 10 |
| Peak Size Reject (counts): | 200000000 |
| Smoothing: | Mean 3 Point Smooth |
| Spike Threshold Factor: | 10 |
| Noise: | Peak to Peak |

| | Retention Time | Area | % of Total | Signal/Noise | Scan Description |
|---|---|---|---|---|---|
| 1. | 2.700 | 3.679e+8 | 1.642 | 65.66 | Merged |
| 2. | 2.995 | 5.231e+8 | 2.335 | 95.76 | Merged |
| 3. | 18.815 | 2.129e+10 | 95.006 | 1.353e+004 | Merged |
| 4. | 30.684 | 2.279e+8 | 1.017 | 167.5 | Merged |

**Figure 11**. GC-MS analysis results of the reaction product between 1-Chloro-4-Iodobenzene and triphenyltin chloride

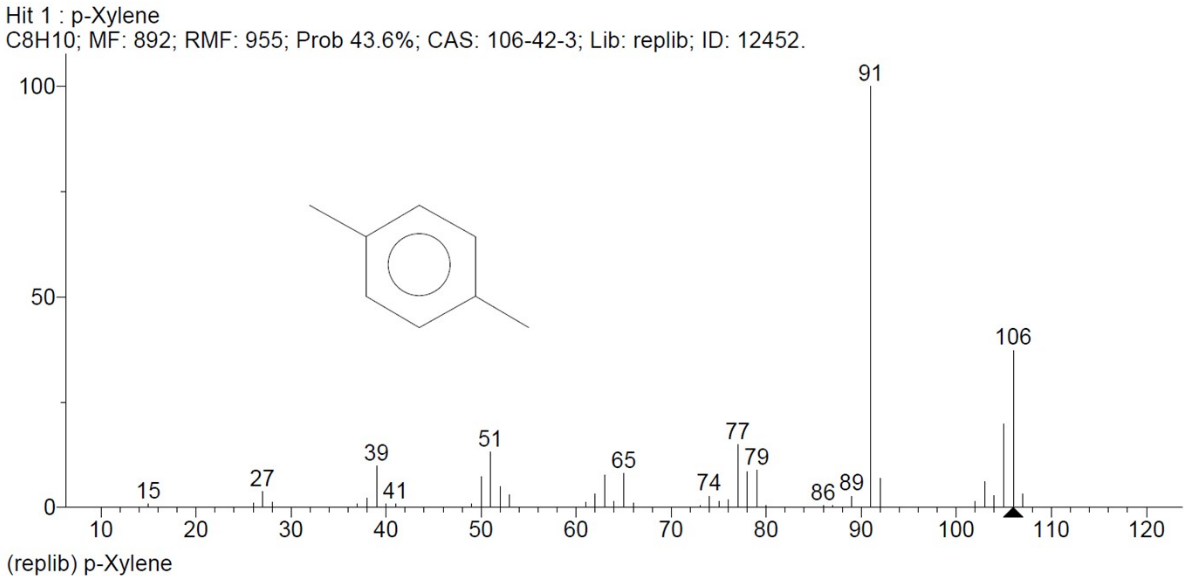


**Figure 11-1.** Retention time 2.700

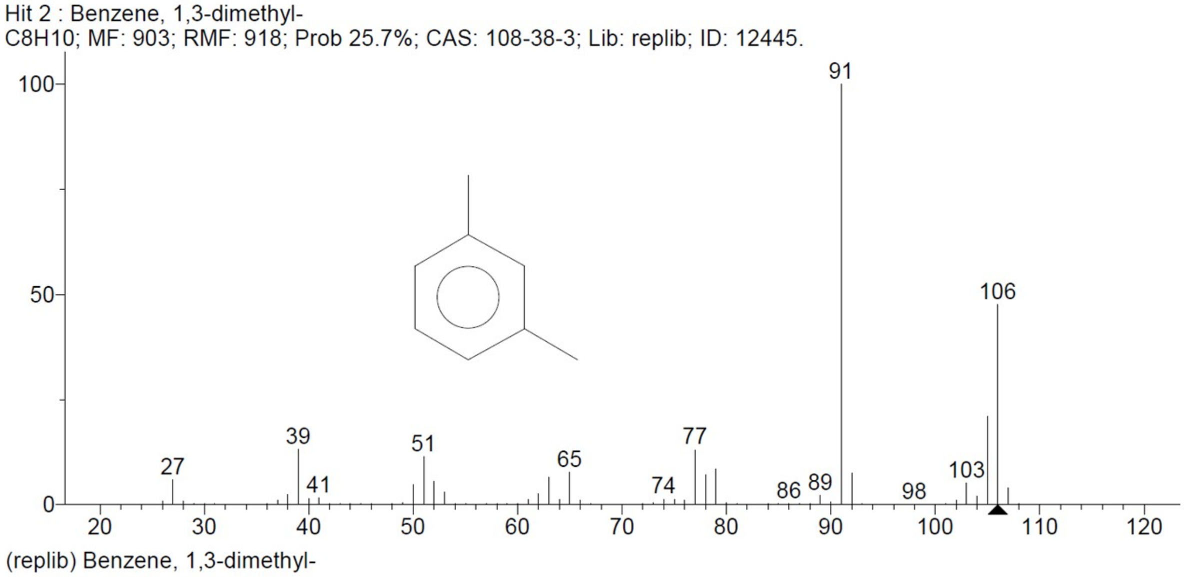


**Figure 11-2.** Retention time 2.995

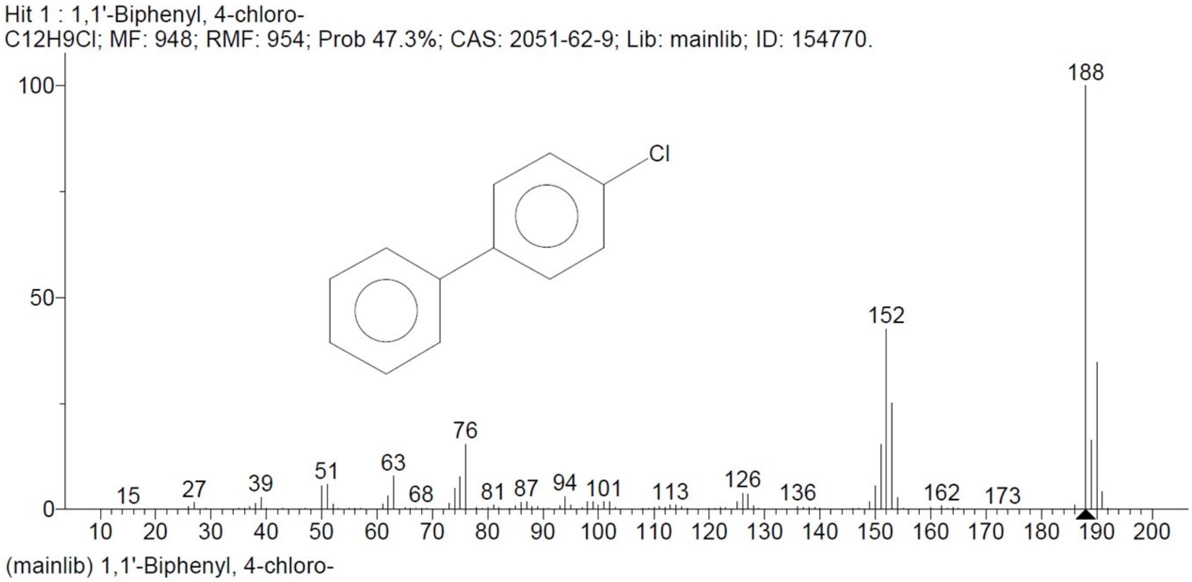


**Figure 11-3.** Retention time 18.815, major product

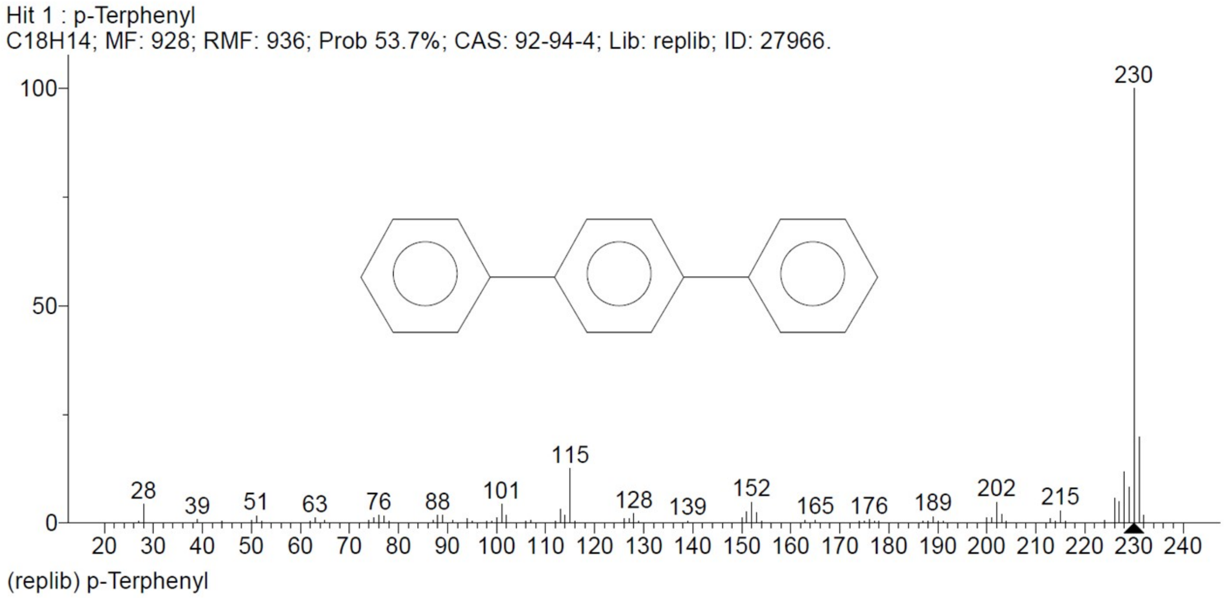


**Figure 11-4.** Retention time 30.684

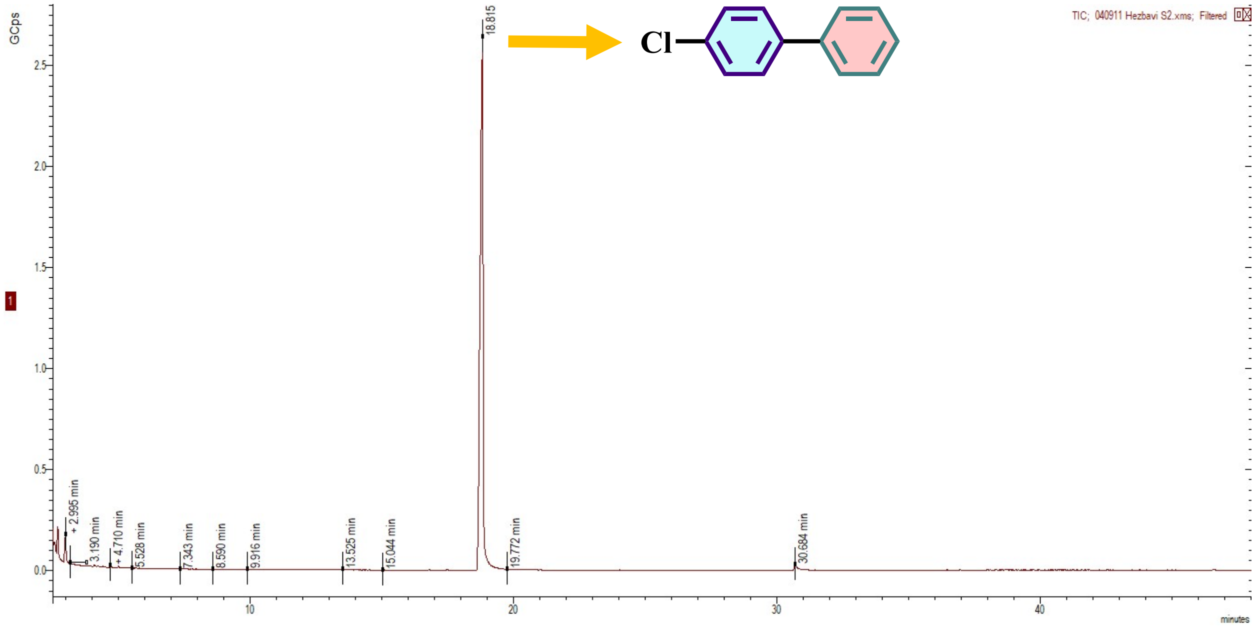


**Figure 12.** GC-MS of the reaction product between 1-Chloro-4-Iodobenzene and triphenyltin chloride: (4-Chloro-1,1'-biphenyl )
